# Implementation and Clinical Adoption of Precision Oncology Workflows Across a Healthcare Network

**DOI:** 10.1093/oncolo/oyac134

**Published:** 2022-07-19

**Authors:** Dora Dias-Santagata, Rebecca S Heist, Adam Z Bard, Annacarolina F L da Silva, Ibiayi Dagogo-Jack, Valentina Nardi, Lauren L Ritterhouse, Laura M Spring, Nicholas Jessop, Alexander A Farahani, Mari Mino-Kenudson, Jill Allen, Lipika Goyal, Aparna Parikh, Joseph Misdraji, Ganesh Shankar, Justin T Jordan, Maria Martinez-Lage, Matthew Frosch, Timothy Graubert, Amir T Fathi, Gabriela S Hobbs, Robert P Hasserjian, Noopur Raje, Jeremy Abramson, Joel H Schwartz, Ryan J Sullivan, David Miller, Mai P Hoang, Steven Isakoff, Amy Ly, Sara Bouberhan, Jaclyn Watkins, Esther Oliva, Lori Wirth, Peter M Sadow, William Faquin, Gregory M Cote, Yin P Hung, Xin Gao, Chin-Lee Wu, Salil Garg, Miguel Rivera, Long P Le, A John Iafrate, Dejan Juric, Ephraim P Hochberg, Jeffrey Clark, Aditya Bardia, Jochen K Lennerz

**Affiliations:** Department of Pathology, Massachusetts General Hospital, Harvard Medical School, Boston, MA, USA; Massachusetts General Hospital Cancer Center, Harvard Medical School, Boston, MA, USA; Department of Pathology, Massachusetts General Hospital, Harvard Medical School, Boston, MA, USA; Department of Pathology, Brigham and Women’s Hospital, Harvard Medical School, Boston, MA, USA; Massachusetts General Hospital Cancer Center, Harvard Medical School, Boston, MA, USA; Department of Pathology, Massachusetts General Hospital, Harvard Medical School, Boston, MA, USA; Department of Pathology, Massachusetts General Hospital, Harvard Medical School, Boston, MA, USA; Massachusetts General Hospital Cancer Center, Harvard Medical School, Boston, MA, USA; Department of Pathology, Massachusetts General Hospital, Harvard Medical School, Boston, MA, USA; Department of Pathology, Massachusetts General Hospital, Harvard Medical School, Boston, MA, USA; Department of Pathology, Massachusetts General Hospital, Harvard Medical School, Boston, MA, USA; Massachusetts General Hospital Cancer Center, Harvard Medical School, Boston, MA, USA; Massachusetts General Hospital Cancer Center, Harvard Medical School, Boston, MA, USA; Massachusetts General Hospital Cancer Center, Harvard Medical School, Boston, MA, USA; Department of Pathology, Massachusetts General Hospital, Harvard Medical School, Boston, MA, USA; Present affiliation: Department of Pathology, Yale University, New Haven, CT, USA; Department of Neurosurgery, Massachusetts General Hospital, Harvard Medical School, Boston, MA, USA; Massachusetts General Hospital Cancer Center, Harvard Medical School, Boston, MA, USA; Department of Pathology, Massachusetts General Hospital, Harvard Medical School, Boston, MA, USA; Department of Pathology, Massachusetts General Hospital, Harvard Medical School, Boston, MA, USA; Massachusetts General Hospital Cancer Center, Harvard Medical School, Boston, MA, USA; Massachusetts General Hospital Cancer Center, Harvard Medical School, Boston, MA, USA; Massachusetts General Hospital Cancer Center, Harvard Medical School, Boston, MA, USA; Department of Pathology, Massachusetts General Hospital, Harvard Medical School, Boston, MA, USA; Massachusetts General Hospital Cancer Center, Harvard Medical School, Boston, MA, USA; Massachusetts General Hospital Cancer Center, Harvard Medical School, Boston, MA, USA; Massachusetts General Hospital Cancer Center, Harvard Medical School, Boston, MA, USA; Massachusetts General Hospital Cancer Center, Harvard Medical School, Boston, MA, USA; Massachusetts General Hospital Cancer Center, Harvard Medical School, Boston, MA, USA; Department of Pathology, Massachusetts General Hospital, Harvard Medical School, Boston, MA, USA; Massachusetts General Hospital Cancer Center, Harvard Medical School, Boston, MA, USA; Department of Pathology, Massachusetts General Hospital, Harvard Medical School, Boston, MA, USA; Massachusetts General Hospital Cancer Center, Harvard Medical School, Boston, MA, USA; Department of Pathology, Massachusetts General Hospital, Harvard Medical School, Boston, MA, USA; Department of Pathology, Massachusetts General Hospital, Harvard Medical School, Boston, MA, USA; Massachusetts General Hospital Cancer Center, Harvard Medical School, Boston, MA, USA; Department of Pathology, Massachusetts General Hospital, Harvard Medical School, Boston, MA, USA; Department of Pathology, Massachusetts General Hospital, Harvard Medical School, Boston, MA, USA; Massachusetts General Hospital Cancer Center, Harvard Medical School, Boston, MA, USA; Department of Pathology, Massachusetts General Hospital, Harvard Medical School, Boston, MA, USA; Massachusetts General Hospital Cancer Center, Harvard Medical School, Boston, MA, USA; Department of Pathology, Massachusetts General Hospital, Harvard Medical School, Boston, MA, USA; Department of Pathology, Massachusetts General Hospital, Harvard Medical School, Boston, MA, USA; Department of Pathology, Massachusetts General Hospital, Harvard Medical School, Boston, MA, USA; Department of Pathology, Massachusetts General Hospital, Harvard Medical School, Boston, MA, USA; Department of Pathology, Massachusetts General Hospital, Harvard Medical School, Boston, MA, USA; Massachusetts General Hospital Cancer Center, Harvard Medical School, Boston, MA, USA; Massachusetts General Hospital Cancer Center, Harvard Medical School, Boston, MA, USA; Massachusetts General Hospital Cancer Center, Harvard Medical School, Boston, MA, USA; Massachusetts General Hospital Cancer Center, Harvard Medical School, Boston, MA, USA; Department of Pathology, Massachusetts General Hospital, Harvard Medical School, Boston, MA, USA

**Keywords:** utilization management, companion diagnostics, targeted therapy, biomarkers

## Abstract

**Background:**

Precision oncology relies on molecular diagnostics, and the value-proposition of modern healthcare networks promises a higher standard of care across partner sites. We present the results of a clinical pilot to standardize precision oncology workflows.

**Methods:**

Workflows are defined as the development, roll-out, and updating of disease-specific molecular order sets. We tracked the timeline, composition, and effort of consensus meetings to define the combination of molecular tests. To assess clinical impact, we examined order set adoption over a two-year period (before and after roll-out) across all gastrointestinal and hepatopancreatobiliary (GI) malignancies, and by provider location within the network.

**Results:**

Development of 12 disease center-specific order sets took ~9 months, and the average number of tests per indication changed from 2.9 to 2.8 (*P* = .74). After roll-out, we identified significant increases in requests for GI patients (17%; *P* < .001), compliance with testing recommendations (9%; *P* < .001), and the fraction of “abnormal” results (6%; *P* < .001). Of 1088 GI patients, only 3 received targeted agents based on findings derived from non-recommended orders (1 before and 2 after roll-out); indicating that our practice did not negatively affect patient treatments. Preliminary analysis showed 99% compliance by providers in network sites, confirming the adoption of the order sets across the network.

**Conclusion:**

Our study details the effort of establishing precision oncology workflows, the adoption pattern, and the absence of harm from the reduction of non-recommended orders. Establishing a modifiable communication tool for molecular testing is an essential component to optimize patient care via precision oncology.

## Teaser

This article presents the results of a clinical pilot to standardize precision oncology workflows across a healthcare network. The authors describe an interdisciplinary approach that can be mirrored in other institutions to reflect particular needs or reframed and applied to other clinical scenarios.

**Implications for Practice**

Realization of precision oncology relies on having *all relevant* molecular test results available for patient management. Best practices entail disease type and stage-based matching of the right tests to the right patients. Establishing and maintaining best practices across a multi-site healthcare network poses unique challenges. We present the development, roll-out, and initial adoption pattern of a low-cost utilization management tool that establishes best practices for molecular test orders. Our data indicate adoption without unintended harm caused by the reduction of non-recommended tests. The presented approach aligns patient and oncologist interests by means of access to best practice recommendations.

## Introduction

**Figure 1.**
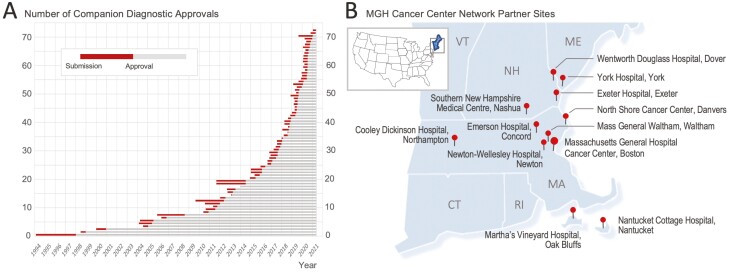
Implementation of precision oncology in a healthcare network. **A.** Overview of number of FDA-approved companion diagnostics over time. Note the rapidly increasing number of approvals, with more than half of all companion diagnostics being approved in the last 3 years. Each row represents a biomarker for a specific drug in a specific indication (i.e., cancer setting). The red bars indicate that the variability in FDA review times (from submission to approval) has stabilized. Details including the biomarker, treatment name, cancer type, and the submission and approval dates are provided in Supplementary Table S2. **B.** The map shows an overview of the MGH Cancer Center affiliates in our healthcare network in New England (blue, inset). Note that the network crosses state-borders, which poses certain operational challenges.

Cancer genotyping has become part of the standard of care in oncology.^1,2^ Treatment options evolve, and numerous medical societies and professional organizations govern the process of incorporating new findings into updated guidelines.^3-7^ For example, the NCCN guidelines continue to evolve over time and recommend combinations of tests for a series of tumors (e.g., PD-L1 + *EGFR* + *ALK* + *ROS1* + *RET* + *MET* exon 14 skipping + *KRAS* G12C + *BRAF* in advanced-stage non-small cell lung cancer).^8,9^ New regulatory paradigms have resulted in increased efficiency of the Food and Drug Administration (FDA) review process.^10,11^ As a result, the number of authorized therapies that rely on biomarkers (i.e., companion diagnostics) has increased sharply in recent decades and currently comprises over 70 approvals (Fig. 1A).^12^ Concomitantly, the number of oncology clinical trials is growing, but many protocols fail to complete because they do not meet their enrollment targets,^13^ at least in part due to lack of broad-scale testing.^14^ More importantly, patients may miss out on a potentially beneficial clinical trial therapy because they were not tested broadly for certain biomarkers.^15,16^ In addition, racial and socio-economic disparities affect the quality of cancer care for many patients, compromising access to biomarker testing and clinical trial enrollment.^17^ Despite substantial progress, realizing and maintaining precision oncology continues to rely on performing the right tests for the right patient at the right time.

Healthcare networks promise improved care by harmonizing access to experts and medical services across the system while simultaneously reducing redundant administrative overhead.^18-20^ The realization of this promise requires careful coordination and consideration of social determinants to identify gaps and provide equity-oriented care.^20,21^ In the context of precision oncology, this coordination entails the integration of various molecular tests.^7^ Importantly, the complexity and cost-coverage of these tests are associated with significant administrative hurdles (e.g., prior authorization, sample selection, result interpretation, and denial management). Overcoming these administrative challenges while accounting for progress in the field requires novel approaches.^22^ There are several commercial solutions including streamlining of test orders and remote testing,^2^ prior authorization,^23^ and trial matching.^24^ However, these solutions rely heavily on local pathology and/or information technology (IT) services that include documentation, data sharing, and frequent status updates.^18,19^ Furthermore, the value-proposition of these additional cost components (overhead) relies largely on longer-term outcome measures that require separate initiatives and efforts for identification and tracking.^25^ To our knowledge, a straightforward and cost-efficient approach to communicate harmonized molecular test combinations, for realizing precision oncology across sites, has not been established.

Here we report the implementation and clinical adoption of precision oncology workflows across a healthcare network. This included the development, roll-out, and continuous improvements of molecular order sets for appropriate test selection by the disease center. We examined adoption patterns across all gastrointestinal malignancies over a two-year period. The frequency of new discoveries in precision oncology and the complexity of molecular test combinations underscore the urgent need for efficient and continuously updated clinical decision support mechanisms. A cost-cognizant communication tool across disease centers is an essential component for the effective delivery of precision oncology to cancer patients.

## Methods

### Project Design and Setting

The project was designed as a prospective quality improvement initiative including a retrospective chart review. The prospective component of the project aimed to optimize clinical test order practice and did not require formal review or approval by the institutional review board (IRB, Human Research Committee, version 25th May 2012). IRB approval was obtained for retrospective chart review (IRB 2008-P-002165). Cancer care was coordinated as a subspecialized tertiary care practice that includes inpatient, outpatient, and a network of community-based sites. All laboratory tests were performed in CLIA-certified laboratories. The molecular laboratory offered 41 high-complexity *in vitro* diagnostic tests^26^ and for certain tests (e.g., HER2, PD-L1, and MMR), immunohistochemistry (IHC)-based assays were included.^27-29^ Prior authorization, consent management, cost estimation, and appeal workflows were managed by the lab, in close coordination with various hospital-based groups.

### Order Sets and Definitions

*Order set* refers to a combination of recommended tests by disease type and setting (e.g., tumor type, stage, and grade). Order sets were designed by an organ- or disease-center-specific *working group* composed of molecular pathologists, medical oncologists, and subspecialty experts from surgical pathology. The working groups considered FDA-approved agents with companion diagnostic designation as well as relevant biomarkers mentioned in professional guidelines (e.g., NCCN, and cIMPACT).^30,31^ The order set design also accounted for recent tumor-agnostic FDA approvals for immune checkpoint inhibitors (ICI). Based on the package insert for each agent and disease setting (Supplementary Table S1 illustrates the analysis for pembrolizumab), we added the relevant biomarker(s) including PD-L1 (IHC), MMR (IHC), and tumor mutational burden status (TMB, provided by the NGS-based mutational analysis/snapshot assay). Furthermore, we included biomarkers with emerging evidence (i.e., peer-reviewed evidence)^32^ after considering applicable governmental and private payor policies. We excluded all research-based biomarker testing. In disease settings for which more than one biomarker-guided therapy may be relevant (FDA-approval or emerging evidence), comprehensive testing using next generation sequencing-based multi-gene panels is recommended (refer to Supplementary Appendices S1 and S2 for more information on the cancer gene panels).^7^ For anatomic figures in order sets we used BioRender.com. For the design of each order set, we tracked the start date, number of and hours in meetings, first approved version date, and go-live date. We also compared the rate of order set adoption after rollout, by the site.

### Data Analysis

For *companion diagnostics review* (Fig. 1A) we performed ongoing monthly checks of FDA announcements and pulled data from the FDA’s medical device database.^33^ We extracted names of the companion diagnostic tests, biomarker, test-type, cancer type (if applicable), submission and approval date, as well as the relevant treatment (status 3/22/2021; Supplementary Table S2).

For *clinical impact analysis,* we used the first (gastrointestinal) order set and compared the year before (2/1/2017–1/31/2018) to the year after roll-out (2/1/2018–1/31/2019). We pulled data from our laboratory information system using a customized Python script using the pandas, NumPy, and Matplotlib libraries. For every ordered test we assigned a “recommended” vs. “non- recommended” label based on whether a specific test was part of the order set that the multidisciplinary panelists had agreed upon for that disease and stage, or not, respectively.

We defined *clinical adoption* as the total number (or fraction) of recommended orders. Notably, the analysis was restricted to requests submitted to our molecular laboratory. We compared the total number of tests, the average number of tests per order, and the number of tests by primary site. Test results were assigned either a “normal” or “abnormal” status. All variants were classified following consensus recommendation^34^ and for all “abnormal” results we assigned one of three labels: non-actionable finding, potentially actionable finding not used for patient management, and actionable findings resulting in targeted treatment, including clinical trial enrollment (Supplementary Fig. S1); “actionable” indicates an association between the molecular diagnostic finding and sensitivity (or resistance) to treatment with a specific FDA-authorized drug (either for that tumor, or for another cancer indication), or the possibility of enrollment in a clinical trial, specific to that tumor and molecular alteration. The assumption was that the implementation of molecular order sets would reduce the number of non-recommended tests. However, to formally investigate unintentional harm due to discouragement of ordering certain tests, we performed a chart review of all non-recommended orders and (after exclusion of redundant, duplicate, screening, or confirmatory tests; Supplementary Fig. S1) and compared the number of actionable alterations before and after roll-out (Supplementary Fig. S1). Data were analyzed using Prism 9 (GraphPad Software Inc., San Diego, CA, USA) and Microsoft Excel for Mac V16.48 (Microsoft Corp., Redmond WA, USA) and we considered *P* values < .05 as indicative of a statistically significant difference (Supplementary Table S3).

## Results

### Realizing Precision Oncology in a Modern Healthcare Network

**Figure 2.**
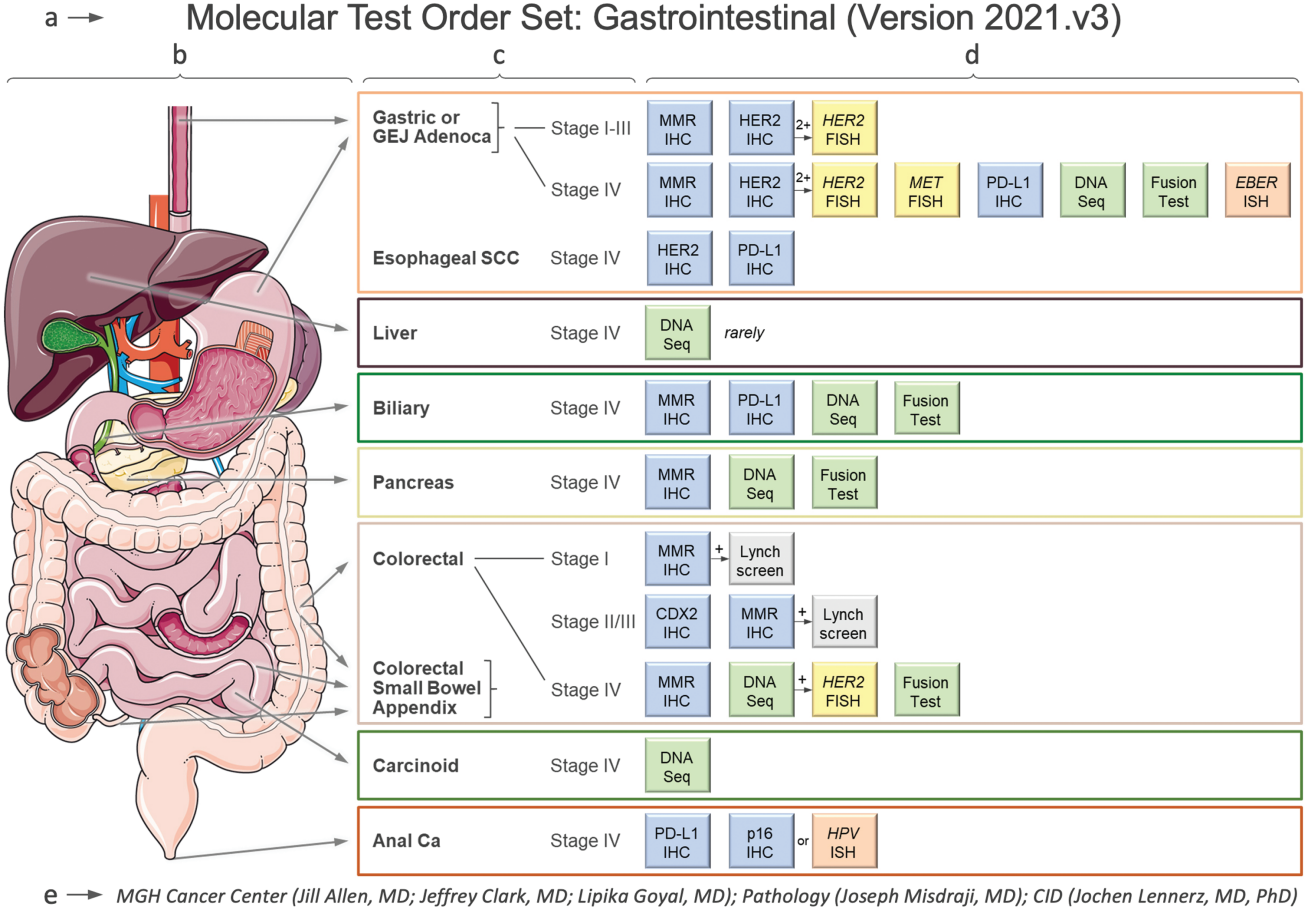
Molecular order set for common gastrointestinal cancers. The test order set consists of five key components: (**a**) the title and version, (**b**) an anatomic scheme that can be used for patient encounters and visual guidance, (**c**) the clinical setting in terms of primary site and stage, and (**d**) the various molecular tests that capture the recommended best practices, established by (**e**) the working group who agreed upon the test order recommendations. The panel can be updated as needed (versioning). The order set includes two comprehensive NGS-based cancer gene panels (in green): a DNA-based assay for the detection of point mutations, indels, and copy number variants (DNA Seq); and an RNA-based assay for the detection of gene rearrangements (Fusion Test). Supplementary Appendix S4 provides our physician order entry form, along with *molecular test order sets* for 12 disease groups, and the proposed workflows for Lynch syndrome screening in patients with GI and uterine cancers. For the latest version of the document, send a blank email to: mghcancersnapshot@partners.org (Supplementary Fig. S3). Abbreviations: Adenoca, adenocarcinoma; Ca, carcinoma; CDX2, caudal-type homeobox transcription factor 2; CID, Center for Integrated Diagnostics; EBER, Epstein-Barr virus-encoded RNA; FISH, fluorescence *in situ* hybridization; GEJ, gastroesophageal junction; GI, gastrointestinal; HER2, erb-b2 receptor tyrosine kinase 2 (*aka* ERBB2); HPV, human papillomavirus; IHC, immunohistochemistry; ISH, *in situ* hybridization; MET, *MET* proto-oncogene, receptor tyrosine kinase (*aka* hepatocyte growth factor receptor); MGH, Massachusetts General Hospital; MMR, mismatch Repair; NGS, next generation sequencing; p16: cyclin-dependent kinase inhibitor 2A (*aka* CDKN2A and p16^INK4a^); PD-L1, programmed death-ligand 1; SCC, squamous cell carcinoma.

To improve access to precision oncology in a complex healthcare system (Fig. 1), a basic requirement for effective patient management is the standardization of molecular testing recommendations across affiliated sites. To address test order harmonization, we optimized molecular ordering for gastrointestinal (GI) malignancies. In a collaboration between molecular pathology, gastrointestinal oncology, and surgical pathology, we created a “GI Molecular Order Set” (Fig. 2). The order sets include anatomic schemes that can be used for patient encounters and visual guidance. It outlines the most relevant clinical settings (i.e., primary site and stage) and lists the molecular tests indicated for each individual condition. The molecular order sets reflect our best practices and have evolved over time (current version: 2021.v3).

### Implementation of a Molecular Order Set for Common Gastrointestinal Cancers

**Figure 3.**
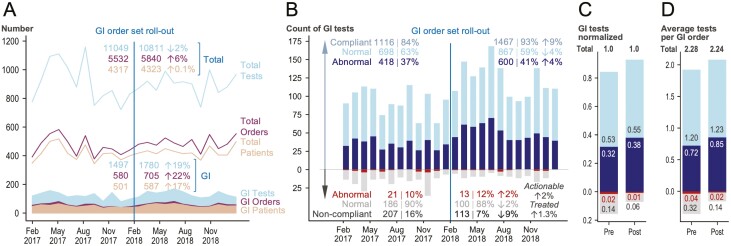
Timeline and clinical impact analysis of the gastrointestinal (GI) order set. **A**. Volumes before and after order set roll-out. There is a significantly higher number of GI patients, accompanied by a significant rise in GI orders and in GI tests when compared to our overall lab growth over the same period. **B.** Bar graph depicts the changes in GI tests before and after order set roll-out. There is a significant increase in recommended tests and “abnormal” test results, indicating adoption by the ordering providers and an increased chance of identifying tumors with “abnormal” (potentially relevant) results, respectively. To assess whether the perceived restrictions on non-recommended orders had a negative effect on the identification and treatment of patients with actionable findings, we looked at the numbers of actionable results and targeted treatments, within the subset of non-recommended orders, and found them to be virtually unchanged (i.e., no negative impact in patient care). In this panel, the fractions of normal and “abnormal” findings were calculated separately, for each subset of recommended or non-recommended results. For a description of test selection for the clinical impact analysis, and for a detailed breakdown of “abnormal” vs. actionable results, and treated patients, please refer to Supplementary Fig. S1. **C.** Test results calculated as a fraction of all GI tests, before and after order set roll-out, showed a considerable rise in recommended requests and in “abnormal” findings. **D.** Number of tests per order showed a minimal increase with a shapeshift of increasing recommended and decreasing non-recommended tests per order. Note: A, raw values; B–D, revised values, after a manual review of non-recommended GI tests (blue boxes in Supplementary Fig. S1).

The first order set was officially launched in February 2018. At that point, when submitting a GI cancer order to the molecular diagnostics lab, clinicians could simply choose “Panel Testing,” to request the designated list of recommended molecular tests for each cancer type/stage. It is worth noting that panel testing was not enforced; it was offered as a simplified, “one-click,” efficient alternative to selecting individual tests, but clinicians still had the option to order tests of their choice. After the GI order set roll-out, we experienced changes in our total volumes (Fig. 3A). By comparison, we noted a significant increase in the number of patients with GI malignancies referred to molecular testing (17%; *P* = .006), accompanied by significant increases in GI orders for testing (22%; *P* =.008) and in actual GI molecular tests (19%; *P* < .001) (Fig. 3A).

### Clinical Adoption and Change in Test Order Practice

To evaluate the impact of the GI order set, we compared the molecular requests submitted to our lab 1 year before and 1 year after roll-out and show that the initiative had an immediate impact on the fraction of recommended tests, which rose from 84% to 93% (Fig. 3B). We consider the high baseline rate of compliance with recommended tests indicative of oncologist expertise, and the overall 9% increase in recommended test orders as a net positive impact of the initiative. Conversely, the effect on non-recommended orders was a reduction from 16% to 7% (Fig. 3B). The observed shift in order practice was statistically significant (*P* < .001), and consistent with clinical adoption of the order sets. Although assessment of the adoption across specific network sites was not the primary goal of the initiative, examination by site showed adoption of the order sets in at least 7 network sites (Fig 1B). Specifically, the fraction of recommended orders was significantly higher in the network (99%) when compared to orders from providers primarily practicing at the main campus (92%; *P* = .005).

To improve patient outcomes across the network, the implementation of best practice testing recommendations should, ideally, increase actionable findings and broaden therapeutic options. While the assessment of patient management decisions and clinical trial enrollments fall outside the scope of this report, we examined the rates of “abnormal” test results, as a surrogate for potentially relevant findings. In our cohort, 39% of recommended tests yielded an “abnormal” test result (i.e., any positive finding), while only 11% of non-recommended tests were “abnormal” (Supplementary Fig. S1). The overall fraction of “abnormal” findings was significantly higher in the tests recommended by the guidelines (*P* < .001), suggesting that the order set design appropriately enriched for potentially relevant molecular assays that consequently uncovered clinically significant findings. After order set roll-out, we observed a 4% rise in “abnormal” findings for recommended tests and a 2% rise for “abnormal” findings in non-recommended tests (Fig. 3B). While we cannot prove that this shift was due to the order sets, there was a combined 6% increase in “abnormal” results (*P* = .002).

### No Significant Impact in the Administration of Molecularly Matched Therapies Driven by Non-Recommended Orders

Unintended consequences of utilization management are of key concern. Specifically, a utilization management strategy may result in unintended harm due to the discouragement of providers to order certain tests and subsequent failure to identify actionable results.^35^ We therefore performed a full chart review of all non-recommended tests, to look for potentially actionable findings, and screened for molecularly informed treatments initiated by non-recommended test results. We mapped the distribution of non-recommended tests according to cancer type, assay type, actionability, and treatment decisions (Supplementary Fig. S2). Over the two-year period, 9% of non-recommended tests yielded potentially actionable results (*n* = 18 before and *n* = 12 after; Supplementary Fig. S1). When considering all molecular requests (*n* = 1323 before, *n* = 1580 after, shaded boxes in Supplementary Fig. S1), the fraction of non-recommended tests with actionable findings dropped by 0.6% (from 1.4% to 0.8%). Within the subset of non-recommended tests (*n* = 207 before, *n* = 113 after, Supplementary Fig. S1), the proportion of actionable findings increased by 2% (from 9% to 11%; Fig. 3B; *P* = .55). Notably, a total of 3 patients (*n* = 1 before, *n* = 2 after) received treatment based on actionable findings detected by non-recommended tests (Supplementary Figs. S1 and S2B). Specifically, one patient received an off-label prescription, and two patients enrolled in clinical trials. We thereby confirmed that the fraction of non-recommended tests with actionable findings that resulted in patient treatment was not significantly affected by order set roll-out (*P* = .55; Supplementary Table S3). It increased by 0.06% (from 0.07% to 0.13%) when considering all tests, and by 1.3% (from 0.5% to 1.8 %; Fig. 3B) within the subset of non-recommended tests.

### A Multidisciplinary Approach to Develop Best Order Practices for Precision Oncology

**Figure 4.**
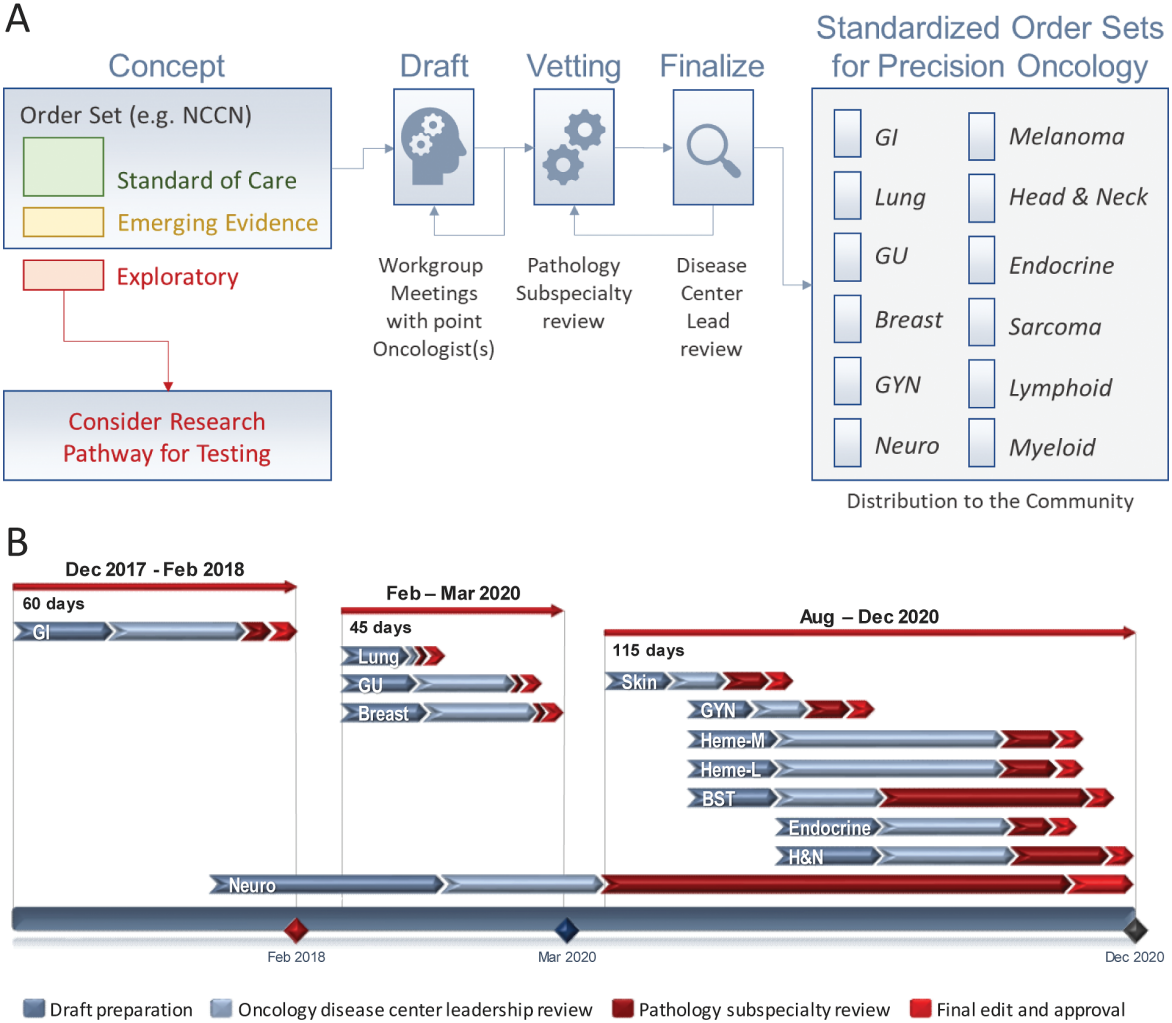
Process design for disease-specific molecular test order sets. **A**. In the concept stage, a molecular pathologist and expert oncologist drafted a list of recommended molecular tests for the most common clinical settings and malignancies. We assigned proposed tests to either *standard of care* (e.g., endorsed by national and international guidelines, and/or testing associated with FDA-approved agents or companion diagnostics; Fig. 1), *emerging evidence* (e.g., peer-reviewed evidence in publications with outcome relevance or clinical trial results) or *exploratory* (i.e., unproven indications and/or research) categories and, for clinical rollout, *exploratory* testing was excluded. The draft was presented in subspecialty workgroup meetings, and once finalized, it was shared and discussed with surgical pathology subspecialty team leaders for review (vetting). For distribution, we standardized the format for physician order entry in the electronic medical record (EMR) and via e-mail for practices and providers outside our EMR. Order sets are updated on a rolling basis when needed (e.g., with new regulatory approvals, guideline changes, etc.). **B.** Simplified Gantt chart for twelve order sets by disease center (i.e., oncologic subspecialties). Each row reflects the actual timeline from drafting to approval. Note the variance of different predecessor tasks between different order sets (e.g., formalizing WHO-based integrated neuropathological diagnosis vs. established genotyping program in the lung); critical paths, meetings, and slack lines are not depicted for simplicity. Supplementary Appendix S4 includes all 12 order sets at the time of publication. Abbreviations: BST, bone and soft tissue cancers; GI, gastrointestinal cancers; GU, genitourinary cancers; GYN, gynecological cancers; Heme-L, hematopoietic and lymphoid malignancies; Heme-M, hematopoietic and myeloid malignancies; H&N, head and neck cancers; NCCN, National Comprehensive Cancer Network (used as an example of widely referenced clinical guidelines).

Based on our experience with the GI order set, we extended the approach and developed molecular order sets for all major cancer indications. The multidisciplinary design followed the same approach as outlined for GI (Fig. 4A) and was conducted in two sprints: order sets for lung, breast, and GU cancers (released in March 2020), and the remaining indications (launched in December 2020; Fig. 4B). We noticed differences in the amount of time required for each order set (range 3–40 h). For example, the order set for neuro-oncology (10 meetings, ~40 h) had to account for the integrated diagnostic paradigm of morphology and molecular findings. Overall, the development of the 12 order sets took approximately 9 months, requiring 0.2 full-time equivalent molecular faculty support, and 42 roundtable discussions. The order sets are provided online (Supplementary Appendix S4), and the latest version may be retrieved by sending a blank email to: mghcancersnapshot@partners.org (Supplementary Fig. S3).

## Discussion

Here we report the clinical adoption of a low-cost approach to harmonize test orders in precision oncology. Precision oncology relies on more than genotyping and next-generation sequencing (NGS) panels. We devised an approach that harnesses the interdisciplinary domain-expertise into a streamlined clinical-decision support tool for molecular test orders. One advantage of our approach to capture the existing expertise is cost-effectiveness and we were not restricted by costly demands, such as hiring new personnel or investing in expensive software solutions. We present a comparison of two 12-months periods, before and after order set implementation across all gastrointestinal malignancies, and we provide the order sets for several disease centers. Importantly, we demonstrate that the approach does not reduce the number of relevant findings in a subset of 9 gastrointestinal cancers. By sharing our multi-year, order optimization initiative, we aim to encourage other healthcare networks to streamline precision oncology orders.

**Figure 5.**
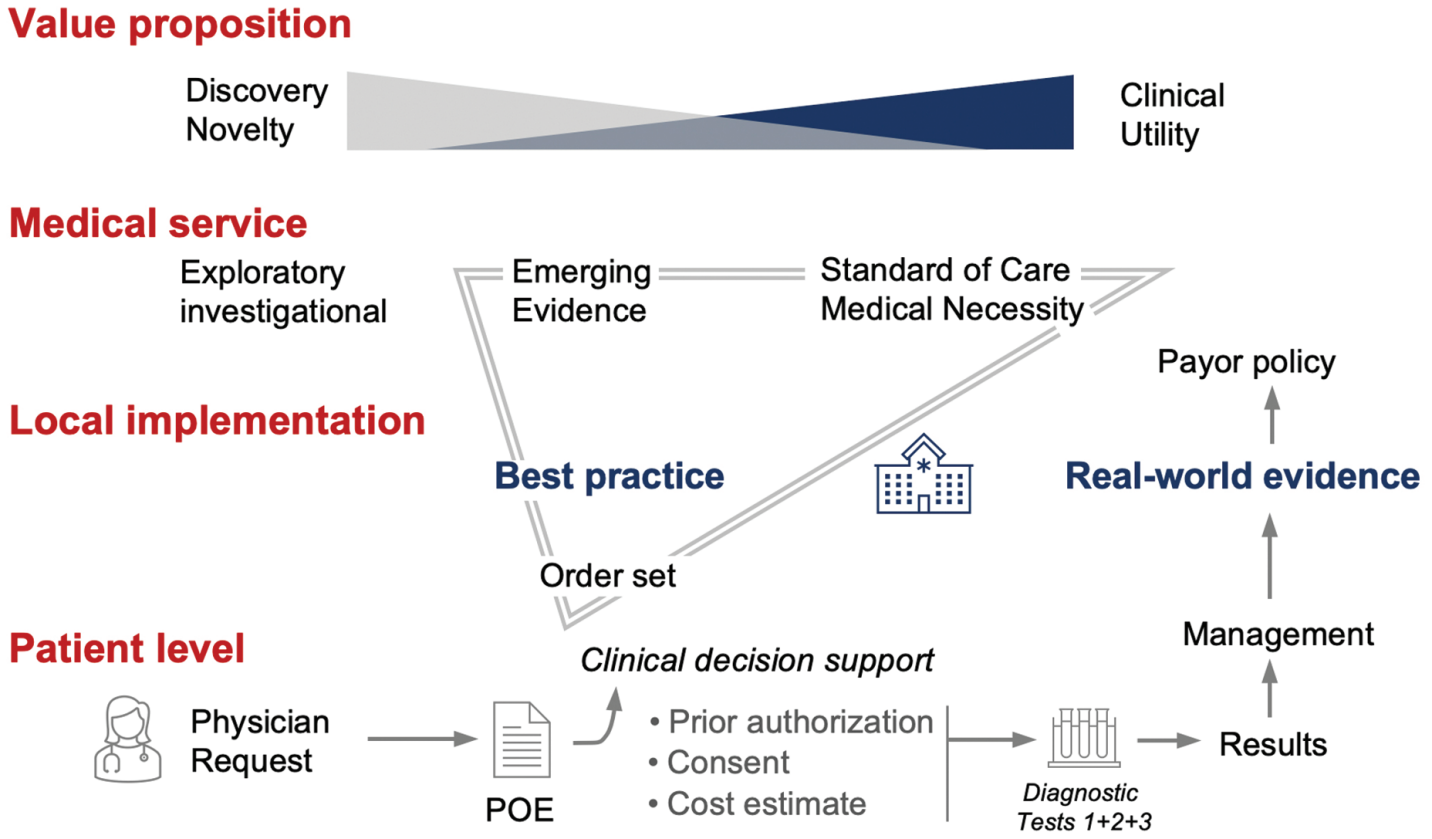
Realizing precision oncology. Precision oncology relies on cutting edge molecular diagnostics. As new discoveries promise additional breakthroughs, the realization at the medical service level must balance emerging evidence and standard of care. We captured best practices by the organ center in the order sets. These collaboratively developed and adaptable lookup tables enable the incorporation of novel recommendations (e.g., from national guidelines, or new FDA approvals), while empowering clinicians by providing clinical decision support. Order sets evolve over time and thereby help incorporate real-world evidence into payor negotiations.

Realizing precision oncology relies on results from more than one test—even more than one panel.^7^ Even the most comprehensive *genotyping* approaches cannot deliver the PD-L1 protein expression status,^27^ the MGMT promoter methylation status in brain tumors,^36,37^ the ER/PR/HER2/Ki-67 labeling index in breast cancer,^38,39^ or cover interdisciplinary Lynch syndrome protocols using mismatch repair protein staining.^28,40^ These are some examples that underscore that precision oncology requires a disease-specific testing approach to obtain the status for all relevant biomarkers—depending on the setting (FDA intended use). Molecularly informed therapies have been associated with improved survival in patients with advanced cancer^41,42^; and improving the availability and timing of pertinent information to assess relevant treatment options has been a central element of numerous initiatives.^43,44^ Furthermore, precision oncology relies on the ongoing incorporation of newly approved treatments, NCCN guideline updates, payor policies, and overcoming administrative hurdles (Fig. 5). Despite systematic attempts,^45,46^ for individual patients, providers face several challenges. For example, access to relevant (molecular-genetic) domain knowledge can be limited,^47,48^ access to so-called best practices remains highly variable, documentation is challenging, and numerous computational solutions have been proposed.^49,50^

Several strategies are integral to practicing precision oncology. These include tumor boards,^51,52^ interdisciplinary consultations, and trial matching—which can, depending on the trial portfolio cause substantial challenges.^15, 53-56^ These various strategies rely in part on molecular test results and here we focused on harmonizing the ordering process for several reasons. First, all patients with advanced cancer and medically relevant indications should be offered testing, regardless of tumor type. The concerted efforts of the NCI-MATCH master trial showed that comprehensive testing across tumor histologies can identify a significant number of actionable alterations and led to successful patient accrual to 30 treatment subprotocols, 11 of which reached their accrual goals.^57^ Second, testing algorithms are complex and depend on tumor stage and type (Supplementary Appendix S4), and recent national data stress the need to guide and educate physicians and patients in the field of genomic profiling.^58^ Third, there is an unrecognized complexity in aligning the various payor policies and guideline recommendations with local test availability. The presented order sets can establish local best practices to harmonize uniform patient access (i.e., access to the right tests for the right patients). However, the realization of the order sets differed in complexity (Supplementary Appendix S4). For example, in neuro-oncology many molecular tests are performed to reach the final diagnosis (i.e., diagnostic biomarkers). Other sets contain a mixture of predictive and diagnostic biomarker considerations (e.g., to distinguish benign from malignant soft tissue tumors, or identify the presence of an *EGFR* activating mutation in a poorly differentiated tumor with equivocal immunophenotype). The creation and implementation of the order sets created a platform that aligns different biomarker functions.^58^ We caution that capturing the vantage points and domain expertise of diverse stakeholders ultimately relies on collaborative synergy.

The limitations of our study are primarily related to our approach. Order set dissemination was done based on the list of providers, and we allowed a combination of EMR- and non-EMR-based order entries, but we could not be certain we reached all providers. This was a deliberate choice to perform short iterative improvements and focus on sustained inter-connectedness of colleagues with domain knowledge from oncology and pathology. Despite the rather simple dissemination, the content was co-created, and the higher rate of recommended orders in network sites can be taken as evidence that the oncologists used the order sets. While we cannot prove causality, the providers were not aware of being observed when ordering. We did not enforce the use and providers continued to be able to order a-la-carte tests and we did not cancel non-recommended orders (i.e., maintained provider preference). Thus, our findings likely reflect the unbiased order practice when offered the local best practice vs. relying on personal experience. A second limitation is that we focused on one organ system (GI) for this analysis. While our GI order set covered 9 different malignancies and 10 different high-complexity assays, it does not cover the entire cohort of GI malignancies. Third, our analysis was limited to the orders that were received by our laboratory. Therefore, we were unable to account for missed tests (i.e., tests that were not ordered, but that should have been run based on the GI order set recommendations). Analysis of non-recommended tests with actionable findings revealed that most requests (15/17) consisted of FISH assays for samples that also underwent (recommended) NGS testing (Supplementary Table S4 and Appendix S3). One reason for these orders is that NGS has lower sensitivity to detect copy number gains especially when the sample has low tumor purity. For example, most commercial labs have a 20% tumor cutoff for molecular assays. Fourth, circulating tumor DNA (ctDNA) analysis was not systematically performed for GI malignancies during the time frame selected for analysis (before 2019). However, our molecular test order sets include ctDNA recommendations for lung, breast, and thyroid to assess disease progression on therapy and/or when obtaining a tissue biopsy is clinically contraindicated (Supplementary Appendix S4). Fifth, we did not perform a detailed financial analysis accounting for the various payors, prior authorization changes, the evolution of payor policies, and professional guidelines over time. We did, however, adjust for volume increases in the laboratory and we consider the shift towards recommended tests with the elimination of non-recommended tests as optimized (net-neutrality; Fig. 3D). However, the appropriate metrics for measuring success are unclear.^59,60^ There is a substantial attrition rate from the initial order to the result, to the identification of the appropriate indication (for FDA-approved agents) or to identifying eligibility for a clinical trial, and finally, the actual delivery of the agent to the patient (Supplementary Fig. S1). Treatment decisions depend on the overall clinical picture *and* on the correct interpretation of the molecular results. Patient management strategies are discussed at subspecialty-specific weekly tumor boards, and unusual cases of general interest are discussed at a weekly consensus meeting and at a monthly molecular tumor board conference. These longstanding conferences include providers across the network and have remained essentially unchanged over the time frame covered by our study (Supplementary Fig. S4). Overcoming practical and administrative hurdles while avoiding disparities and achieving consistent access for all patients requires alignment of seemingly discrepant workflow elements (Fig. 5). We consider harmonizing test order practice an essential element of realizing precision oncology.

The next steps in our precision diagnostics program include three specific ongoing projects. First, we aim to incorporate the ability for e-consultations to serve as a point of contact for network sites interested in exploring reasons for, or against, certain tests. The service line, which was rolled out in February 2022, entails pathology “curbside” consultations in our electronic medical record—and for more comprehensive e-consultations we are using the recently revised clinical-pathological consultation billing framework.^61,62^ Second, we will expand the distribution of our molecular order sets. We consider harmonization of test order practice a key element of equitable care, with uniform access to all, including minorities and those living in rural areas.^63,64^ Third, we plan to incorporate our real-time clinical trial landscape so that it will be available for all providers.

In summary, the implementation of precision oncology workflows relies on numerous elements. Here, we reported a cost-efficient strategy to align test order practices across multiple stakeholders. Standardized test order practice and access to continuously updated domain knowledge are an essential part of precision diagnostic laboratories’ value proposition in an integrated healthcare network.

## Supplementary Material

oyac134_suppl_Supplementary_Information_1

oyac134_suppl_Supplementary_Information_2

## Data Availability

The data underlying this article will be shared on reasonable request to the corresponding author.
