# Supplementary material for: Implementation and Clinical Adoption of Precision Oncology Workflows Across a Healthcare Network
Source: Oncologist. 2022 Jul 19;27(11):930–9. doi: 10.1093/oncolo/oyac134 (PMC9632318; doi:10.1093/oncolo/oyac134)
Supplement: oyac134_suppl_Supplementary_Information_1 [file oyac134_suppl_supplementary_information_1.pdf]

## Supplemental Material

# Implementation and Clinical Adoption of Precision Oncology Workflows Across a Healthcare Network.

Dora Dias-Santagata[1], Rebecca S. Heist[2], Adam Z Bard[1], Annacarolina F. L. da Silva[3], Ibiayi Dagogo-Jack[2], Valentina Nardi[1], Lauren L. Ritterhouse[1], Laura M. Spring[2], Nicholas Jessop[1], Alexander A. Farahani[1], Mari Mino-Kenudson[1], Jill Allen[2], Lipika Goyal[2], Aparna Parikh[2], Joseph Misdraji[1*], Ganesh Shankar[4], Justin T. Jordan[2], Maria Martinez-Lage[1], Matthew Frosch[1], Timothy Graubert[2], Amir T. Fathi[2], Gabriella Hobbs[2], Robert P. Hasserjian[1], Noopur Raje[2], Jeremy Abramson[2], Joel H. Schwartz[2], Ryan J. Sullivan[2], David Miller[2], Mai P. Hoang[1], Steven Isakoff[2], Amy Ly[1], Sara Bouberhan[2], Jaclyn Watkins[1], Esther Oliva[1], Lori Wirth[2], Peter M. Sadow[1], William Faquin[1], Gregory M. Cote[2], Yin P. Hung[1], Xin Gao[2], Chin-Lee Wu[1], Salil Garg[1], Miguel Rivera[1], Long P. Le[1], A. John Iafrate[1], Dejan Juric[2], Ephraim P. Hochberg[2], Jeffrey Clark[2], Aditya Bardia[2], Jochen K. Lennerz[1]

[1]Department of Pathology, Massachusetts General Hospital, Harvard Medical School, Boston, MA, USA; [2]Massachusetts General Hospital Cancer Center, Harvard Medical School, Boston, MA, USA; [3]Department of Pathology, Brigham and Women's Hospital, Harvard Medical School, Boston, MA, USA; [4]Department of Neurosurgery, Massachusetts General Hospital, Harvard Medical School, Boston, MA, USA.
*Current affiliation: Department of Pathology, Yale University, New Haven, CT, USA.

## Content


| | | |
|---|---|---|
| Supplemental Table 1 | Assessment of testing requirements for pembrolizumab……….......... | 2 |
| Supplemental Appendix 1 | Content of the NGS DNA-based Mutational Analysis Assays…………… | 3 |
| Supplemental Appendix 2 | Content of the NGS RNA-based Fusion Assays……………………….…… | 5 |
| Supplemental Table 2 | Companion diagnostics information collected from the FDA medical device database……….………………………………………………….... | 7 |
| Supplemental Figure 1 | GI test selection for the clinical impact analysis.…………………………. | 8 |
| Supplemental Table 3 | Statistical analysis.………………………………………………………… | 9 |
| Supplemental Figure 2 | Distribution of GI molecular requests…………………………………….. | 10 |
| Supplemental Figure 3 | Molecular order sets retrieval………………....…………………………. | 11 |
| Supplemental Table 4 | Non-recommended tests with potentially actionable findings……………. | 12 |
| Supplemental Appendix 3 | Analysis of actionable findings………............................................... | 13 |
| Supplemental Figure 4 | Representative monthly schedule for Molecular Diagnostics and GI oncology conferences.…………………………………………………..... | 14 |

**Supplemental Table 1.** Assessment of testing requirements for pembrolizumab.

| Cancer Type | Setting | Option | Comment |
|---|---|---|---|
| Melanoma | Approved (even adjuvant) | ✓ | |
| | [Data in BRAF mutant as well]* | ✓ | |
| NSCLC | Absence of EGFR/ALK as combination therapy | ✓ | |
| | PD-L1 is neg, Keytruda approved in second line | ✓ | |
| | **[“first line” desirable but not in package insert]** | | |
| SCLC | Keytruda approved in second line | ✓ | |
| | TMB-high approval not relevant because already approved | ✓ | data in package insert (Table 59) |
| HNSCC | PD-L1 is high = approved | ✓ | |
| | PD-L1 is 0 = in second line second line | ✓ | |
| cHL | approved in second line | ✓ | |
| | TMB-high approval not relevant because already approved | | |
| | [genotyping is currently not reliably achievable in clinical routine] | | |
| PMBL | approved in second line | ✓ | |
| | TMB-high approval not relevant because already approved | | |
| Urothelial | approved in relapse/progression | ✓ | |
| | TMB-high approval not relevant because already approved | | |
| MSI-H/MMR-def | approved | ✓ | |
| | TMB-high approval not relevant because already approved | | |
| Colorectal | MSI-L/MMRcompetent | ✓ | |
| | TMB may matter in second line | | no data in package insert |
| | [very rare setting that TMB high and MMRcompetent, e.g. POLD/POLE] | | comment |
| PDAC | MSI-L/MMRcompetent | ✓ | |
| | TMB may matter in second line | | |
| Gastric | PD-L1 + and 3rd line = Keytruda approved | ✓ | |
| | in PD-L1 neg and 2nd line, TMB high = Keytruda | | no data in package insert |
| Esophagel | PD-L1 + and 2nd line = Keytruda approved | ✓ | |
| | in PD-L1 neg and 2nd line, TMB high = Keytruda | | no data in package insert |
| Cervical Cancer | PD-L1 + and 2nd line = Keytruda approved | ✓ | |
| | in PD-L1 neg and 2nd line, TMB high = Keytruda | ✓ | data in package insert (Table 59) |
| HCC | second line (with prior sorafenib) | ✓ | |
| | TMB may matter if the first line was not sorafenib and recurrence | | no data in package insert |
| Merkel Cell | approved for recurrence/second line | ✓ | |
| | TMB-high approval not relevant because already approved | | |
| Renal | axitinib + Keytruda first line | ✓ | |
| | TMB-high approval not relevant because already approved | | |
| Endometrial | MSI-H => approved | ✓ | |
| | TMB-high approval not relevant because already approved | | |
| | MSI-low, approved in second line | ✓ | |
| | TMB-high approval not relevant because already approved | | |
| Cut Squam | approved for recurrence | ✓ | |
| | TMB-high approval not relevant because already approved | | |
| **Rare Tumors** | | | |
| Anal Cancer | TMB may matter in second line | ✓ | data in package insert (Table 59) |
| Vulvar Cancer | TMB may matter in second line | ✓ | data in package insert (Table 59) |
| Neuroendocrine | TMB may matter in second line | ✓ | data in package insert (Table 59) |
| Salivary gland | TMB may matter in second line | ✓ | data in package insert (Table 59) |
| Thyroid Cancer | TMB may matter in second line | ✓ | data in package insert (Table 59) |
| Mesothelioma | TMB may matter in second line | | data in package insert (Table 59) |

Note: based on data in the package insert for Keytruda (2019).

**Supplemental Appendix 1. Content of the NGS DNA-based Mutational Analysis Assays**

**SNAPSHOT-NGS-V2 Assay**

Genes (exons) targeted for SNV and indel assessment: *ABL1* (4-7), *AKT1* (3,6), *ALK* (21-23,25), *APC* (16), *ARID1A* (1-20), *ATM* (1-63), *ATRX* (1-35), *AURKA* (2,5-8), *BRAF* (11,15), *BRCA1* (2-23), *BRCA2* (2-27), *CCNE1* (3-8,10,12), *CDH1* (1-16), *CDK4* (2-7), *CDKN2A* (1-3), *CIC* (1-20), *CSF1R* (7,22), *CTNNB1* (3), *DAXX* (1-8), *DDR2* (12-18), *DDX3X* (1-17), *EGFR* (3,7,15,18-21), *ERBB2* (8,10,19-21,24), *ERBB3* (2-3,7-8), *ERBB4* (3-4,6-9,15,23), *ESR1* (8), *EZH2* (16), *FBXW7* (1-11), *FGFR1* (4,7-8,13,15,17), *FGFR2* (7,9,12,14), *FGFR3* (7-9,14-16,18), *FLT3* (11,14,16,20), *FOXL2* (1), *GNA11* (5), *GNAQ* (4-5), *GNAS* (6-9), *H3F3A* (2), *HNF1A* (3-4), *HRAS* (2-3), *IDH1* (3-4), *IDH2* (4), *JAK2* (11,13-14,16,19), *JAK3* (4,13,16), *KDR* (6-7,11,19,21,26-27,30), *KEAP1* (2-6), *KIT* (2,8-11,13-15,17-18), *KRAS* (2-5), *MAP2K1* (2,3,6-7), *MAP3K1* (1-20), *MDM2* (2-4,6,8,10), *MEN1* (2-10), *MET* (2,11,14,16,19,21), *MLH1* (12), *MPL* (10), *MSH6* (1-10), *MSI*, *MYC* (1-3), *MYCN* (3), *NF1* (1-58), *NF2* (1-15), *NOTCH1* (25-27,34), *NPM1* (11), *NRAS* (2-5), *PIK3CA* (2,5,7-8,10,14,19,21), *PIK3R1* (1-10), *POLE* (9-14), *PTCH1* (1-23), *PTEN* (1-9), *PTPN11* (3,13), *RB1* (1-27), *RET* (10-11,13-16), *RHOA* (2-3), *RNF43* (2-10), *ROS1* (38), *SDHB* (1-8), *SMAD2* (7), *SMAD4* (2-12), *SMARCA4* (3-36), *SMARCB1* (2,4,5,9), *SMO* (3,5-6,9,11), *SRC* (14), *STAG2* (3-34), *STK11* (1-9), *SUFU* (1-12), *TERT* (1), *TP53* (1-11), *TP63* (1-14), *TSC1* (3-23), *TSC2* (2-42), *TSHR* (10), and *VHL* (1-3).

Genes targeted for CNV assessment: *ABL1, AKT1, ALK, APC, ARID1A, ATM, ATRX, AURKA, BRAF, BRCA1, BRCA2, CAMTA1, CCNB1, CCND1, CCND2, CCND3, CCNE1, CDK4, CDKN2A, CDK6, CIC, CDH1, CSF1R, DAXX, DDR2, DDX3X, EGFR, ERBB2 (HER-2), ERBB3, ERBB4, FBXW7, FGF19, FGFR1, FGFR2, FGFR3, FLT3, FOXL2, GLI2, GNA11, GNAQ, GNAS, HNF1A, HRAS, IDH1, JAK2, JAK3, KDR, KEAP1, KIT, KRAS, MAP2K1, MAP3K1, MDM2, MDM4, MEN1, MET, MITF, MLH1, MSH6, MYC, MYCN, NF1, NF2, NKX2-1, NOTCH1, NRAS, PDGFRA, PIK3CA, PIK3R1, PLAUR, POLE, PTCH1, PTEN, PTPN11, RB1, RET, RHOA, RNF43, SDHB, SMAD2, SMAD4, SMARCA4, SMARCB1, SMO, SRC, STAG2, STK11, SUFU, TERT, TP53, TP63, TSC1, TSC2,* and *VHL.*

**Heme SNAPSHOT-NGS-V4 Assay**

Targeted genes (exons): *ABL1* (4-10), *ALK* (22-25), *ANKRD26* (1), *ARID1A* (1-20), *ASXL1* (1-12), *ATM* (1-63), *ATRX* (8-11,17-32), *BCL2* (2), *BCOR* (2-15), *BCORL1* (1-12), *BCR* (1-5), *BIRC3* (2-9),

*BRAF* (3,10-15), *BTK* (15), *CALR* (1-9), *CARD11* (5-9), *CBL* (2-5,7-9,16), *CBLB* (3,9-10), *CBLC* (9-10), *CCND2* (5), *CCR4* (2), *CD79A* (4-5), *CD79B* (5-6), *CDKN2A* (1-3), *CEBPA* (1), *CREBBP* (1-31), *CSF3R* (10,14-18), *CUX1* (1-24), *CXCR4* (1-2), *DCK* (2-3), *DDX41* (1-17), *DHX15* (3), *DNM2* (17,19), *DNMT3A* (1-23), *EP300* (1-31), *ETNK1* (3), *ETV6* (1-8), *EZH2* (2-20), *FBXW7* (1-11), *FLT3* (8-17,19-21), *FOXO1* (1), *GATA1* (2), *GATA2* (2-6), *GNAS* (8-11), *HRAS* (2-4), IDH1 (3-4), *IDH2* (4,6), *IKZF1* (2-6, del 1-3), *IKZF3* (5,8), *JAK1* (14-16), *JAK2* (12-16,19-25), *JAK3* (3,11,13,15,18,19), *KDM5A* (8,11,13-14,18,21,23,25), *KDM6A* (1-29), *KIT* (1-2,5,8-15,17,18), *KLF2* (1-3), *KMT2A* (1-36), *KMT2C* (14,25,27,36,38,43-44,55), *KMT2D* (8,11,15,31,34,39,44,53), *KMT2E* (14-15,21), *KRAS* (2-4), *LUC7L2* (1-10), *MAP2K1* (1-11), *MEF2B* (1-2), *MPL* (10,12), *MYC* (1-3), *MYD88* (3-5), *NF1* (1-57), *NFKBIE* (1), *NOTCH1* (UTR,26-28,34), *NOTCH2* (34), *NPM1* (11), *NRAS* (2-5), *NT5C2* (9,11,13,15,17-19), *PDGFRA* (12,14,15,18), *PHF6* (2-10), *PLCG2* (19,24), *PML* (1-9), *PPM1D* (6), *PRPF40B* (1-26), *PTEN* (1-9), *PTPN11* (3-4,7-8,11-13), *RAD21* (2-14), *RARA* (5-7,9), *RB1* (1-27), *RBBP6* (16,18), *RHOA* (2), *RPS15* (4), *RUNX1* (2-9), *SETBP1* (4), *SETD2* (1-21), *SF3B1* (13-21), *SH2B3* (2-8), *SLC29A1* (4,13), *SMC1A* (1-25), *SMC3* (10,13,19,23,25,28), *SRC* (10), *SRSF2* (1-2), *STAG2* (2-33), *STAT3* (2-24), *STAT5B* (15-17), *STAT6* (5,12), *TET2* (3-11), *TNFAIP3* (1-9), *TNFRSF14* (1-6), *TP53* (1-11), *U2AF1* (2,6-7), *U2AF2* (1-12), *WT1* (1-9), *XPO1* (15-16,18), and *ZRSR2* (1-11).

## Supplemental Appendix 2. Content of the NGS RNA-based Fusion Assays*

### Solid Fusion Assay V2

Targeted genes (exons): *AKT3* (1-3), *ALK* (19-22, intron 19), *AR* (1-8), *ARHGAP26* (2,10-12), *AXL* (19,20), *BRAF* (7-12,15), *BRD3* (9-12), *BRD4* (10,11), *CSF1* (5-9), *CSF1R* (7,11-13,22), *EGFR* (2-7 exon skipping/vIII variant, 7-9,16,20,24,25), *ERG* (2-11), *ESR1* (3-6), *ESRRA* (3-5), *ETV1* (3-13), *ETV4* (2,4-10), *ETV5* (2,3,7-9), *ETV6* (1-7), *EWSR1* (4-14), *FGFR1* (2,8-10,17), *FGFR2* (2,8-10,17), *FGFR3* (8-10,17, intron 17), *FGR* (2), *INSR* (12-22), *JAZF1* (2-4), *MAML2* (2,3), *MAST1* (7-9, 18-21), *MAST2* (2,3,5,6), *MET* (13, exon 14 skipping, 15), *MSMB* (2-4), *MUSK* (7-9,11-14), *MYB* (7-9,11-16), *NOTCH1* (2,4, 26-31, internal exon 3-27 deletion), *NOTCH2* (5-7,26-28), *NRG1* (1-3, 6), *NTRK1* (8,10-13), *NTRK2* (11-17), *NTRK3* (13-16), *NUMBL* (3), *NUTM1* (3), *PDGFRA* (7, exon 8 deletion, 10-14), *PDGFRB* (8-14), *PIK3CA* (2), *PKN1* (10-13), *PPARG* (1-3), *PRKCA* (4-6), *PRKCB* (3), *RAF1* (4-7, 9-12), *RELA* (3,4), *RET* (8-13), *ROS1* (31-37), *RSPO2* (1, 2), *RSPO3* (2), *TERT* (2), *TFE3* (2-8), *TFEB* (1,2), *THADA* (24-31,36), and *TMPRSS2* (1-6).

### Heme Fusion Assay V3

Targeted genes (exons): *ABL1* (1-5), *ABL2* (2-8), *ALK* (2,4,6,10,16-23), *BCL11B* (2-4), *BCL2* (2-3), *BCL6* (2-3), *BCR* (1-3,8,12-16), *BIRC3* (4-7), *BRAF* (7-12,15), *CBFB* (4,5), *CCND1* (5), *CCND3* (2), *CD19* (exon 2 skipping detection), *CD28* (4), *CDK6* (1-4), *CHD1* (1-2), *CHIC2* (1-3), *CIITA* (1-2), *CREBBP* (2-6), *CRLF2* (1-6), *CSF1R* (9-14), *CTLA4* (3), *DEK* (2-3), *DUSP22* (1-2), *EBF1* (10-15), *EIF4A1* (2-3), *ERG* (7-11), *ETV6* (1-6), *FGFR1* (2-12,17), *FLT3* (14-18,20), *GLIS2* (2-3), *IKZF1* (1-3,7-8), *IKZF2* (3-4), *IKZF3* (2-7), *IL2RB* (2), *JAK2* (6-20), *KAT6A* (13-16), *KLF2* (2,3), *KMT2A* (2-35), *LYN* (6,7), *MALT1* (9), *MECOM* (1-4), *MEF2D* (5-8), *MKL1* (4-6), *MLF1* (2-4), *MLLT10* (2-18), *MLLT4* (2), *MYC* (1-2), *MYH11* (7-11,14-16), *NF1* (14,36), *NFKB2* (14-21), *NOTCH1* (24-29,34), *NOTCH2* (26-28), *NOTCH3* (25-27), *NOTCH4* (22-24), *NTRK3* (15), *NUP214* (17-19), *NUP98* (8-17), *P2RY8* (1), *PAG1* (2), *PAX5* (1,4-8), *PDCD1LG2* (1-3,5-6), *PDGFRA* (9-14), *PDGFRB* (8-14), *PICALM* (16-19), *PML* (2-7), *PRDM16* (1-4), *PTK2B* (2-8), *RARA* (2-5), *RBM15* (1), *ROS1* (33-36), *RUNX1* (2-9), *RUNX1T1* (2-3), *SEMA6A* (1-2), *SETD2* (1-12), *STIL* (1-2), *SYK* (3-8), *TAL1* (2-4), *TCF3* (11-18), *TFG* (2-4), *TP63* (3-5), *TSLP* (1), *TYK2* (16-18), *VAV* (24-25), *ZCCHC7* (1-4), and *ZNF384* (2-7).

**Sarcoma Fusion Assay V1**

Targeted genes (exons): *ALK* (19, 20, 21, 22), *CAMTA1* (8,9,10), *CCNB3* (2,3,4,5,6), *CIC* (19,20), *EPC1* (9,10,11), *EWSR1* (3,4,5,6,7,8,9,10,11,12,13), *FOXO1* (1,2,3), *FUS* (4,5,6,7,8,9,10,11,14), *GLI1* (4,5,6,7), *HMGA2* (1,2,3,4,5), *JAZF1* (2,3,4), *MEAF6* (4,5), *MKL2* (11,12,13), *NCOA2* (11,12,13,14), *NTRK3* (13,14,15,16), *PDGFB* (2,3), *PLAG1* (1,2,3,4), *ROS1* (31,32,33,34,35,36,37), *SS18* (4,5,6,8,9,10,11), *STAT6* (1,2,3,4,5,6,7,16,17,18,19), *TAF15* (5,6,7), *TCF12* (4,5,6), *TFE3* (3,4,5,6), *TFG* (4,5,6,7), *USP6* (1,2,3), *YWHAE* (5).

*Reference: Zongli Zheng Z, Matthew Liebers M, Boryana Zhelyazkova B *et al.* Anchored multiplex PCR for targeted next-generation sequencing. Nature Medicine 2014;20:1479–1484.

**Supplemental Table 2.** Companion diagnostics information collected from the FDA medical device database.

| Count | Diagnostic Name | Biomarker | Test | Cancer Type | Submission | Decision | Review ti | Treatment | URL |
|---|---|---|---|---|---|---|---|---|---|
| 1 | BRACAnalysis CDx | BRACAnalysis | DNA | Ovarian | 6/29/2018 | 10/16/2018 | 109 | Rubraca® (rucaparib) | https://www.accessdata.fda.gov/scripts/cdrh/cfdocs/cfpma/pma.cfm?id=P140020S016 |
| 2 | BRACAnalysis CDx | BRACAnalysis | DNA | Pancreas | 7/2/2019 | 12/27/2019 | 178 | LYNPARZA (olaparib) | https://www.accessdata.fda.gov/scripts/cdrh/cfdocs/cfpma/pma.cfm?id=P140020S019 |
| 3 | BRACAnalysis CDx | BRACAnalysis | DNA | mCRPC | 12/6/2019 | 5/19/2020 | 165 | Lynparza® (olaparib) | https://www.accessdata.fda.gov/scripts/cdrh/cfdocs/cfpma/pma.cfm?id=P140020S020 |
| 4 | therascreen EGFR RGQ PCR K | EGFR | PCR | NSCLC | 2/16/2018 | 9/27/2018 | 223 | dacomitinib | https://www.accessdata.fda.gov/scripts/cdrh/cfdocs/cfpma/pma.cfm?id=P120022S018 |
| 5 | cobas EGFR Mutation Test v2 | EGFR | DNA | NSCLC | 2/23/2018 | 8/22/2018 | 180 | TARCEVA® (erlotinib) TAGRIS | https://www.accessdata.fda.gov/scripts/cdrh/cfdocs/cfpma/pma.cfm?id=P120019s019 |
| 6 | PD-L1 IHC 22C3 pharmDx | PD-L1 IHC 22C3 | IHC | NSCLC | 4/6/2015 | 10/2/2015 | 179 | KEYTRUDA (PEMBROLIZUMA | https://www.accessdata.fda.gov/scripts/cdrh/cfdocs/cfpma/pma.cfm?id=P150013 |
| 7 | PD-L1 IHC 22C3 pharmDx | PD-L1 IHC 22C3 | IHC | GEJ | 4/19/2017 | 9/22/2017 | 156 | KEYTRUDA® (pembrolizumab | https://www.accessdata.fda.gov/scripts/cdrh/cfdocs/cfpma/pma.cfm?id=P150013s006 |
| 8 | PD-L1 IHC 22C3 pharmDx | PD-L1 IHC 22C4 | IHC | Cervix | 1/29/2018 | 6/12/2018 | 134 | KEYTRUDA® (pembrolizumab | https://www.accessdata.fda.gov/scripts/cdrh/cfdocs/cfpma/pma.cfm?id=P150013s009 |
| 9 | PD-L1 IHC 22C3 pharmDx | PD-L1 IHC 22C5 | IHC | Urothelial | 6/25/2018 | 8/16/2018 | 52 | KEYTRUDA® (pembrolizumab | https://www.accessdata.fda.gov/scripts/cdrh/cfdocs/cfpma/pma.cfm?id=P150013s011 |
| 10 | PD-L1 IHC 22C3 pharmDx | PD-L1 IHC 22C6 | IHC | Head and neck | 1/18/2019 | 6/10/2019 | 143 | KEYTRUDA® (pembrolizumab | https://www.accessdata.fda.gov/scripts/cdrh/cfdocs/cfpma/pma.cfm?id=P150013s014 |
| 11 | PD-L1 IHC 22C3 pharmDx | PD-L1 IHC 22C3 | IHC | Eso-Sq | 2/25/2019 | 7/30/2019 | 155 | KEYTRUDA® (pembrolizumab | https://www.accessdata.fda.gov/scripts/cdrh/cfdocs/cfpma/pma.cfm?id=P150013s016 |
| 12 | PD-L1 IHC 22C3 pharmDx | PD-L1 IHC 22C3 | IHC | TNBC | 6/29/2019 | 11/13/2020 | 503 | KEYTRUDA® (pembrolizumab | https://www.accessdata.fda.gov/scripts/cdrh/cfdocs/cfpma/pma.cfm?id=P150013s020 |
| 13 | PD-L1 IHC 22C3 pharmDx | PD-L1 IHC 22C3 | IHC | NSCLC | 8/31/2020 | 2/22/2021 | 175 | LIBTAYO (cemiplimab) | https://www.accessdata.fda.gov/scripts/cdrh/cfdocs/cfpma/pma.cfm?id=P150013S021 |
| 14 | Abbott Real*Time* IDH1 | IDH1 | DNA | CML | 12/5/2017 | 7/20/2018 | 227 | TIBSOVO® (ivosidenib) | https://www.accessdata.fda.gov/scripts/cdrh/cfdocs/cfpma/pma.cfm?id=P170041 |
| 15 | MRDx BCR-ABL Test | BCR-ABL monitoring | RT-PCR | CML | 11/13/2017 | 12/22/2017 | 39 | nilotinib | https://www.accessdata.fda.gov/scripts/cdrh/cfdocs/cfpmn/pmn.cfm?ID=K173492 |
| 16 | FoundationOne CDx | EGFR, ALK, BRAF, EF | NGs | multiple | 6/2/2017 | 11/30/2017 | 181 | multiple | https://www.accessdata.fda.gov/scripts/cdrh/cfdocs/cfpma/pma.cfm?id=p170019 |
| 17 | FoundationOne CDx | BRCA1/2 alterations | NGS | ovarian cancer | 9/24/2018 | 7/1/2019 | 280 | LYNPARZA (olaparib) | https://www.accessdata.fda.gov/scripts/cdrh/cfdocs/cfpma/pma.cfm?id=P170019S004 |
| 18 | FoundationOne CDx | PIK3CA C420R, E54 | NGS | Breast | 12/26/2018 | 12/3/2019 | 342 | PIQRAY (alpelisib) | https://www.accessdata.fda.gov/scripts/cdrh/cfdocs/cfpma/pma.cfm?id=P170019S006 |
| 19 | FoundationOne CDx | EGFR exon 19 deleti | NGS | non-small cell lung c | 4/9/2019 | 7/1/2019 | 83 | TAGRISSO (osimertinib) | https://www.accessdata.fda.gov/scripts/cdrh/cfdocs/cfpma/pma.cfm?id=P170019S008 |
| 20 | FoundationOne CDx | MET exon 14 skippi | NGs | non-small cell lung c | 10/17/2019 | 5/6/2020 | 202 | TABRECTA (capmatinib) | https://www.accessdata.fda.gov/scripts/cdrh/cfdocs/cfpma/pma.cfm?id=P170019S011 |
| 21 | FoundationOne CDx | FGFR2 rearrangeme | NGS | cholangiocarcinoma | 10/31/2019 | 4/17/2020 | 169 | PEMAZYRE(pemigatinib) | https://www.accessdata.fda.gov/scripts/cdrh/cfdocs/cfpma/pma.cfm?id=P170019S013 |
| 22 | FoundationOne CDx | HRD | NGS | metastatic castratio | 12/16/2019 | 5/19/2020 | 155 | Lynparza®(olaparib). | https://www.accessdata.fda.gov/scripts/cdrh/cfdocs/cfpma/pma.cfm?id=P170019S015 |
| 23 | FoundationOne CDx | TMB>10Mut/Mb | NGS | multiple | 1/21/2020 | 6/16/2020 | 147 | KEYTRUDA® (pembrolizumab | https://www.accessdata.fda.gov/scripts/cdrh/cfdocs/cfpma/pma.cfm?id=P170019S016 |
| 24 | FoundationOne CDx | NTRK | NGS | multiple | 1/31/2020 | 10/23/2020 | 266 | VITRAKVI® (larotrectinib) | https://www.accessdata.fda.gov/scripts/cdrh/cfdocs/cfpma/pma.cfm?id=P170019S017 |
| 25 | VENTANA ALK (D5F3) CDx As | ALK (D5F3) | IHC | anaplastic lymphom | 6/7/2017 | 11/6/2017 | 152 | XALKORI® (crizotinib) or ZYK | https://www.accessdata.fda.gov/scripts/cdrh/cfdocs/cfpma/pma.cfm?id=P140025S006 |
| 26 | VENTANA ALK (D5F3) CDx As | AKK (D5F3) | IHC | NSCLC | 11/16/2020 | 3/3/2021 | 107 | XALKORI® (crizotinib), ZYKAD | https://www.accessdata.fda.gov/scripts/cdrh/cfdocs/cfpma/pma.cfm?id=P140025S014 |
| 27 | Abbott Real*Time* IDH2 | IDH2 nine IDH2 mu | PCR | AML | 2/2/2017 | 8/1/2017 | 180 | IDHIFA® (enasidenib) | https://www.accessdata.fda.gov/scripts/cdrh/cfdocs/cfpma/pma.cfm?id=p170005 |
| 28 | Praxis Extended RAS Panel | RAS genes [KRAS (e | NGS | CRC | 9/2/2016 | 6/29/2017 | 300 | Vectibix® (panitumumab) | https://www.accessdata.fda.gov/scripts/cdrh/cfdocs/cfpma/pma.cfm?id=p160038 |
| 29 | Oncomine Dx Target Test | BRAF ROS1 EGFR | NGS | MEL, NSCLC | 10/17/2016 | 6/22/2017 | 248 | multiple | https://www.accessdata.fda.gov/scripts/cdrh/cfdocs/cfpma/pma.cfm?id=p160038 |
| 30 | Oncomine Dx Target Test | RET fusions | NGS | NSCLC | 4/1/2020 | 9/4/2020 | 156 | GAVRETO (pralsetinib) | https://www.accessdata.fda.gov/scripts/cdrh/cfdocs/cfpma/pma.cfm?id=p160045s019 |
| 31 | LeukoStrat CDx FLT3 Mutatio | FLT3-ITD, D835 and | PCR | AML | 9/2/2016 | 4/28/2017 | 238 | RYDAPT (midostaurin) | https://www.accessdata.fda.gov/scripts/cdrh/cfdocs/cfpma/pma.cfm?id=p160045 |
| 32 | FoundationFocus CDx*BRCA* A | BRCA1/2 | NGS | ovarian | 6/30/2016 | 12/19/2016 | 172 | Rubraca (rucaparib) | https://www.accessdata.fda.gov/scripts/cdrh/cfdocs/cfpma/pma.cfm?id=p160040 |
| 33 | Vysis CLL FISH Probe Kit | TP53 Chromosom 1 | FISH | CLL | 10/30/2015 | 4/11/2016 | 164 | VENCLEXTA® (venetoclax) | https://www.accessdata.fda.gov/scripts/cdrh/cfdocs/cfpma/pma.cfm?id=p160018 |
| 34 | *KIT* D816V Mutation Detecti | Kit d816v mutation | *PCR* | *mastocytosis* | 11/26/2014 | 12/18/2015 | 387 | gleevec® (imatinib mesylate) | https://www.accessdata.fda.gov/scripts/cdrh/cfdocs/cfhde/hde.cfm?id=375586 |
| 35 | *PDGFRB* FISH for Gleevec Eli | *PDGFRB* | *FISH* | *MDS/MPD* | 11/26/2014 | 12/18/2015 | 387 | gleevec (imatinib mesylate) | https://www.accessdata.fda.gov/scripts/cdrh/cfdocs/cfhde/hde.cfm?id=375585 |
| 36 | cobas KRAS Mutation Test | SEVEN SOMATIC M | DNA | CRC | 10/23/2014 | 5/7/2015 | 196 | ERBITUX (CETUXIMAB) OR W | https://www.accessdata.fda.gov/scripts/cdrh/cfdocs/cfpma/pma.cfm?id=p140023 |
| 37 | therascreen KRAS RGQ PCR K | KRAS | PCR | CRC | 7/25/2011 | 5/23/2014 | 1033 | ERBITUX (CETUXIMAB) | https://www.accessdata.fda.gov/scripts/cdrh/cfdocs/cfpma/pma.cfm?id=p110030 |
| 38 | therascreen KRAS RGQ PCR K | KRAS | PCR | CRC | 7/25/2011 | 5/23/2014 | 1033 | ERBITUX (CETUXIMAB) AND \ | https://www.accessdata.fda.gov/scripts/cdrh/cfdocs/cfpma/pma.cfm?id=p110027 |
| 39 | Dako EGFR pharmDx Kit | EGFR | IHC | CRC | 4/3/2006 | 9/27/2006 | 177 | ERBITUX (CETUXIMAB), OR V | https://www.accessdata.fda.gov/scripts/cdrh/cfdocs/cfpma/pma.cfm?id=P030044s002 |
| 40 | FerriScan | Iron concentration | MRI | non-transfusion-de | 1/2/2013 | 1/23/2013 | 21 | deferasirox therapy | https://www.accessdata.fda.gov/scripts/cdrh/cfdocs/cfpmn/denovo.cfm?ID=DEN130012 |
| 41 | Dako c-KIT pharmDx | CKIT | IHC | GIST | 3/12/2004 | 6/27/2005 | 472 | GLEEVEC/GLIVEC (IMATINIB | https://www.accessdata.fda.gov/scripts/cdrh/cfdocs/cfpma/pma.cfm?id=P040011 |
| 42 | INFORM HER-2/neu | HER2 | FISH | Breast | 2/14/1994 | 12/30/1997 | 1415 | risk stratification | https://www.accessdata.fda.gov/scripts/cdrh/cfdocs/cfpma/pma.cfm?id=p940004 |
| 43 | PathVysion HER-2 DNA Probe | HER2 | FISH | Breast | 6/16/1998 | 12/11/1998 | 178 | adjuvant cyclophosphamide, | https://www.accessdata.fda.gov/scripts/cdrh/cfdocs/cfpma/pma.cfm?id=p980024 |
| 44 | PATHWAY anti-Her2/neu (4B | HER-2 (clone CB11) | IHC | Breast | 12/13/1999 | 11/28/2000 | 351 | HERCEPTIN(R) | https://www.accessdata.fda.gov/scripts/cdrh/cfdocs/cfpma/pma.cfm?id=p990081 |
| 45 | PATHWAY anti-Her2/neu (4B | HER-2 (clone CB11) | IHC | Breast | 2/13/2019 | 5/3/2019 | 79 | Herceptin® (trastuzumab) or | https://www.accessdata.fda.gov/scripts/cdrh/cfdocs/cfpma/pma.cfm?id=p990081S039 |
| 46 | InSite Her-2/neu KIT | HER-2 (clone CB11) | IHC | Breast | 6/22/2004 | 12/22/2004 | 183 | HERCEPTIN (TRASTUZUMAB) | https://www.accessdata.fda.gov/scripts/cdrh/cfdocs/cfpma/pma.cfm?id=p040030 |
| 47 | SPOT-LIGHT HER2 CISH Kit | HER2 | CISH | Breast | 11/3/2005 | 7/1/2008 | 971 | HERCEPTIN (TRASTUZUMAB) | https://www.accessdata.fda.gov/scripts/cdrh/cfdocs/cfpma/pma.cfm?id=p050040 |
| 48 | Bond Oracle HER2 IHC Syster | HER2 | IHC | Breast | 7/22/2009 | 4/18/2012 | 1001 | HERCEPTIN (TRASTUZUMAB) | https://www.accessdata.fda.gov/scripts/cdrh/cfdocs/cfpma/pma.cfm?id=P090015 |
| 49 | HER2 CISH pharmDx Kit | HER2 Chromosome | CISH | Breast | 6/21/2010 | 11/30/2011 | 527 | HERCEPTIN (TRASTUZUMAB) | https://www.accessdata.fda.gov/scripts/cdrh/cfdocs/cfpma/pma.cfm?id=p100024 |
| 50 | INFORM HER2 Dual ISH DNA | HER2 Chromosome | CISH | Breast | 7/12/2010 | 6/14/2011 | 337 | I-HERPCEPTIN (TRASTUZUMA | https://www.accessdata.fda.gov/scripts/cdrh/cfdocs/cfpma/pma.cfm?id=p100027 |
| 51 | INFORM HER2 Dual ISH DNA | HER2 Chromosome | CISH | Breast | 2/13/2019 | 5/3/2019 | 79 | Herceptin® (trastuzumab) or | https://www.accessdata.fda.gov/scripts/cdrh/cfdocs/cfpma/pma.cfm?id=p100027S030 |
| 52 | HercepTest | HER2 | IHC | Gastric GEJ | 11/21/2013 | 11/26/2013 | 5 | HERCEPTIN (TRASTUZUMAB) | https://www.accessdata.fda.gov/scripts/cdrh/cfdocs/cfpma/pma.cfm?id=p980018S018 |
| 53 | HER2 FISH pharmDx Kit | HER2 | FISH | Breast | 1/29/2004 | 5/3/2005 | 460 | HERCEPTIN (TRASTUZUMAB) | https://www.accessdata.fda.gov/scripts/cdrh/cfdocs/cfpma/pma.cfm?id=p040005 |
| 54 | HER2 FISH pharmDx Kit | HER2 | FISH | Gastric GEJ | 4/20/2010 | 10/20/2010 | 183 | HERCEPTIN (TRASTUZUMAB) | https://www.accessdata.fda.gov/scripts/cdrh/cfdocs/cfpma/pma.cfm?id=p040005s005 |
| 55 | HER2 FISH pharmDx Kit | HER2 | FISH | Breast | 12/5/2011 | 6/8/2012 | 186 | PERJETA (PERTUZUMAB) | https://www.accessdata.fda.gov/scripts/cdrh/cfdocs/cfpma/pma.cfm?id=p040005s006 |
| 56 | HER2 FISH pharmDx Kit | HER2 | FISH | Breast | 8/24/2012 | 2/22/2013 | 182 | KADCYLA (ADO-TRASTUZUM | https://www.accessdata.fda.gov/scripts/cdrh/cfdocs/cfpma/pma.cfm?id=p040005s009 |
| 57 | THXID BRAF Kit | BRAFV600E/K | DNA | Melanoma | 7/31/2012 | 5/29/2013 | 302 | DABRAFENIB [TAFINLAR®] | https://www.accessdata.fda.gov/scripts/cdrh/cfdocs/cfpma/pma.cfm?id=p120014 |
| 58 | Vysis ALK Break Apart FISH P | ALK | FISH | NSCLC | 4/1/2011 | 8/26/2011 | 147 | XALKORI CRIZOTINIB) | https://www.accessdata.fda.gov/scripts/cdrh/cfdocs/cfpma/pma.cfm?id=p110012 |
| 59 | Vysis ALK Break Apart FISH P | ALK | FISH | NSCLC | 12/23/2019 | 5/22/2020 | 151 | ALUNBRIG® (brigatinib) | https://www.accessdata.fda.gov/scripts/cdrh/cfdocs/cfpma/pma.cfm?id=P110012S020 |
| 60 | cobas 4800 BRAF V600 Muta | BRAF V600E | DNA | Melanoma | 6/30/2016 | 11/7/2016 | 130 | ZELBORAF® (vemurafenib) | https://www.accessdata.fda.gov/scripts/cdrh/cfdocs/cfpma/pma.cfm?id=P110020S016 |
| 61 | VENTANA PD-L1(SP142) Assa | PD-L1(SP142) | IHC | NSCLC | 6/14/2018 | 7/2/2018 | 18 | TECENTRIQ (atezolizumab) | https://www.accessdata.fda.gov/scripts/cdrh/cfdocs/cfpma/pma.cfm?id=P160002S006 |
| 62 | VENTANA PD-L1(SP142) Assa | PD L1 (SP142) | IHC | Urothelial, TNBC, NS | 9/14/2018 | 3/8/2019 | 175 | TECENTRIQ (atezolizumab) | https://www.accessdata.fda.gov/scripts/cdrh/cfdocs/cfpma/pma.cfm?id=P160002S009 |
| 63 | VENTANA PD-L1(SP142) Assa | PD-L1 (SP142) | IHC | NSCLC | 1/21/2020 | 5/18/2020 | 118 | TECENTRIQ (atezolizumab) | https://www.accessdata.fda.gov/scripts/cdrh/cfdocs/cfpma/pma.cfm?id=P160002S012 |
| 64 | *therascreen* FGFR RGQ RT-P | *FGFR abnormalities* | *DNA* | *Urothelial* | 11/8/2018 | 4/12/2019 | 155 | BALVERSA (erdafitinib) | https://www.accessdata.fda.gov/scripts/cdrh/cfdocs/cfPMA/pma.cfm?id=P180043 |
| 65 | *therascreen* PIK3CA RGQ PC | *PIK3CA* | *DNA* | *Breast* | 1/17/2019 | 5/24/2019 | 127 | PIQRAY (alpelisib) | https://www.accessdata.fda.gov/scripts/cdrh/cfdocs/cfpma/pma.cfm?id=P190001 |
| 66 | *therascreen* PIK3CA RGQ PC | *PIK3CA* | *DNA* | *Breast* | 2/15/2019 | 5/24/2019 | 98 | PIQRAY (alpelisib) | https://www.accessdata.fda.gov/scripts/cdrh/cfdocs/cfPMA/pma.cfm?id=P190004 |
| 67 | Myriad myChoice® CDx | *BRCA1/2* | *NGS* | *ovarian* | 5/3/2019 | 10/23/2019 | 173 | Zejula® (niraparib) | https://www.accessdata.fda.gov/scripts/cdrh/cfdocs/cfpma/pma.cfm?id=P190014 |
| 68 | Myriad myChoice® CDx | *BRCA1/2 (HRD)* | *NGS* | *ovarian maintenanc* | 2/11/2020 | 5/8/2020 | 87 | Lynparza® (olaparib) | https://www.accessdata.fda.gov/scripts/cdrh/cfdocs/cfpma/pma.cfm?id=P190014S003 |
| 69 | *therascreen* BRAF V600E RG | *BRAF V600E* | *DNA* | *CRC* | 9/30/2019 | 4/15/2020 | 198 | BRAFTOVI (encorafenib) | https://www.accessdata.fda.gov/scripts/cdrh/cfdocs/cfpma/pma.cfm?id=P190026 |
| 70 | PD-L1 IHC 28-8 pharmDx | PD-L1 IHC 28-8 | *IHC* | *NSCLC, Sqcell H&N,* | 12/16/2019 | 5/15/2020 | 151 | YERVOY® (ipilimumab) | https://www.accessdata.fda.gov/scripts/cdrh/cfdocs/cfpma/pma.cfm?id=P150025S013 |
| 71 | cobas EZH2 Mutation Test | *EZH2* | *DNA* | *follicular lymphom* | 3/2/2020 | 6/18/2020 | 108 | TAZVERIK (tazemetostat) | https://www.accessdata.fda.gov/scripts/cdrh/cfdocs/cfpma/pma.cfm?id=p200014 |
| 72 | Ventana HER2 Dual ISH DNA | *HER2* | *ISH* | *Breast* | 11/29/2019 | 7/28/2020 | 242 | HERCEPTIN (TRASTUZUMAB) | https://www.accessdata.fda.gov/scripts/cdrh/cfdocs/cfpma/pma.cfm?id=p190031 |
| 73 | Guardant 360® CDx | *EGFR* | *NGS* | *NSCLC* | 2/10/2020 | 8/7/2020 | 179 | Tagrisso (osimertinib) | https://www.accessdata.fda.gov/scripts/cdrh/cfdocs/cfpma/pma.cfm?id=p200010 |

**From:**
https://www.fda.gov/medical-devices/in-vitro-diagnostics/list-cleared-or-approved-companion-diagnostic-devices-in-vitro-and-imaging-tools

**Status:** 3/22/2021

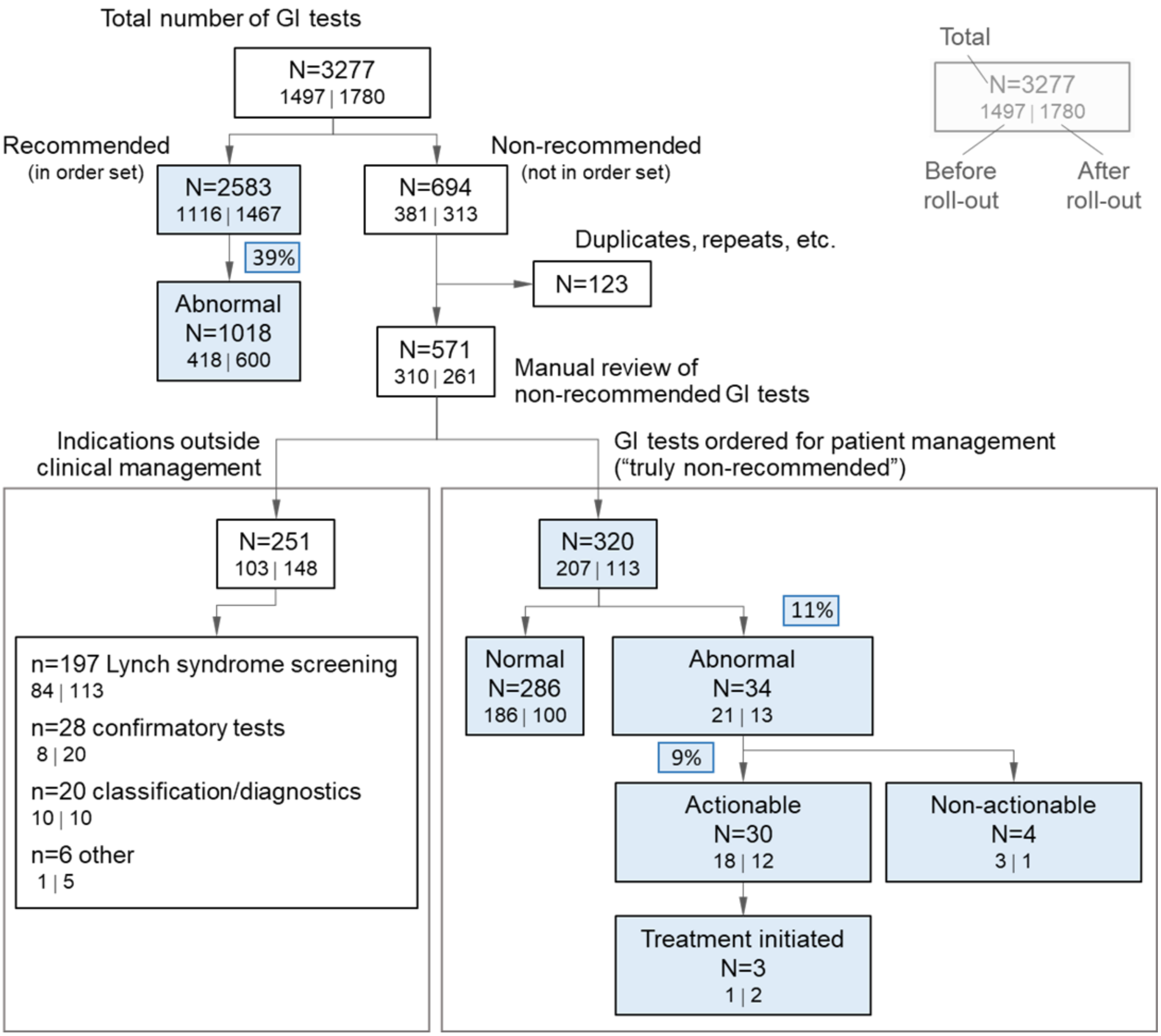


**Supplemental Figure 1. GI test selection for the clinical impact analysis.** Flow diagram summarizing the types of GI molecular tests ordered during the two-years chosen to evaluate the impact of the GI order set. We assessed compliance with order set recommendations. Non-recommended tests were examined in detail. After elimination of duplicates, repeats, and tests ordered for indications outside of clinical management, we identified a subset of tests submitted for patient management (i.e., "truly non-recommended"). For the clinical impact analysis (Fig. 3B), we compared the recommended tests (N=2583) with the subset of "truly non-recommended" tests (N=320), shown in the blue boxes. Results were summarized as normal or abnormal, and as actionable or not actionable. The number of patients treated with targeted agents based on actionable findings obtained by non-recommended tests is also shown.

**Supplemental Table 3.** Statistical analysis.

| | Metric | Fisher's Exact Test Contingency Tables | | P value |
|---|---|---|---|---|
| A | patients (GI vs. non-GI)<br>before vs. after rollout | GI patients/before<br>501 | GI patients/after<br>587 | **P=0.006** |
| | | non-GI patients/before<br>3,816 | non-GI patients/after<br>3,736 | |
| B | order volumes (GI vs. non-GI)<br>before vs. after rollout | GI orders/before<br>580 | GI orders/after<br>705 | **P=0.008** |
| | | non-GI orders/before<br>4,952 | non-GI orders/after<br>5,135 | |
| C | molecular tests (GI vs. non-GI)<br>before vs. after rollout | GI tests/before<br>1,497 | GI tests/after<br>1,780 | **P<0.00001** |
| | | non-GI tests/before<br>9,552 | non-GI tests/after<br>9,031 | |
| D | compliance in GI test requests<br>before vs. after rollout | recommended/before<br>1,116 | recommended/after<br>1,467 | **P<0.00001** |
| | | non-recommended/before<br>207 | non-recommended/after<br>113 | |
| E | compliance in GI test requests after roll-out<br>main campus vs. network sites | recommended, after roll-out/main campus<br>1,363 | recommended, after roll-out/network sites<br>104 | **P=0.005** |
| | | non-recommended, after roll-out/main campus<br>112 | non-recommended, after roll-out/network sites<br>1 | |
| F | GI test results (normal vs abnormal)<br>recommended vs. non-recommended | recommended/normal<br>1,565 (698+867) | recommended/abnormal<br>1,018 (418+600) | **P<0.00001** |
| | | non-recommended/normal<br>286 (186+100) | non-recommended/abnormal<br>34 (21+13) | |
| G | GI test results (normal vs abnormal)<br>before vs. after rollout | abnormal/before<br>439 (418+21) | abnormal/after<br>613 (600+13) | **P=0.002** |
| | | normal/before<br>884 (698+186) | normal/after<br>967 (867+100) | |
| H | actionable results in<br>non-recommended GI tests<br>before vs. after rollout | non-recommended, actionable/before<br>18 | non-recommended, actionable/after<br>12 | P=0.55 |
| | | non-recommended, not actionable/before<br>189 | non-recommended, not actionable/after<br>101 | |
| I | patients treated based on actionable results<br>in non-recommended GI tests<br>before vs. after rollout | non-recommended, actionable, treated/before<br>1 | non-recommended, actionable, treated/after<br>2 | P=0.55 |
| | | non-recommended, actionable, not-treated/before<br>17 | non-recommended, actionable, not-treated/after<br>10 | |

Note: Test selection to evaluate the clinical impact of the GI order set is outlined in Figure S1, and entailed manual review of non-recommended GI tests (to exclude duplicates, repeats and indications outside of clinical management). Statistical analysis: panels A-C include "raw" numbers (i.e. recommended + all non-recommended tests, prior to manual review); panels D-I include "revised" numbers (blue shading in Figure S1: recommended + "truly non-recommended " tests).

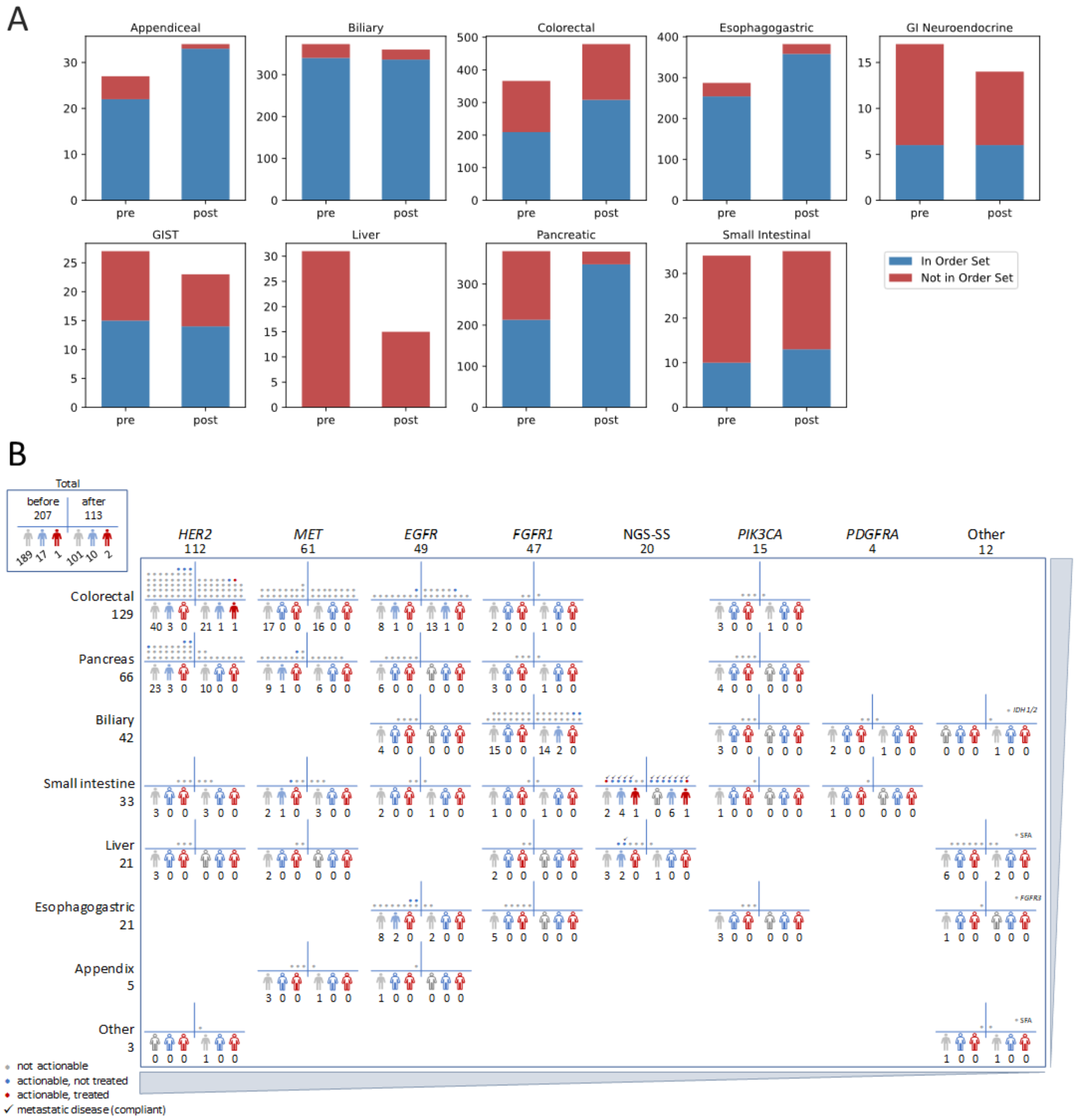


**Supplemental Figure 2. Distribution of GI molecular requests.** A. Total number of GI molecular tests ordered before and after order set implementation, according to cancer site. B. Distribution of non-recommended tests ordered before and after GI order set roll-out, according to assay type, cancer type, actionability, and treatment decisions

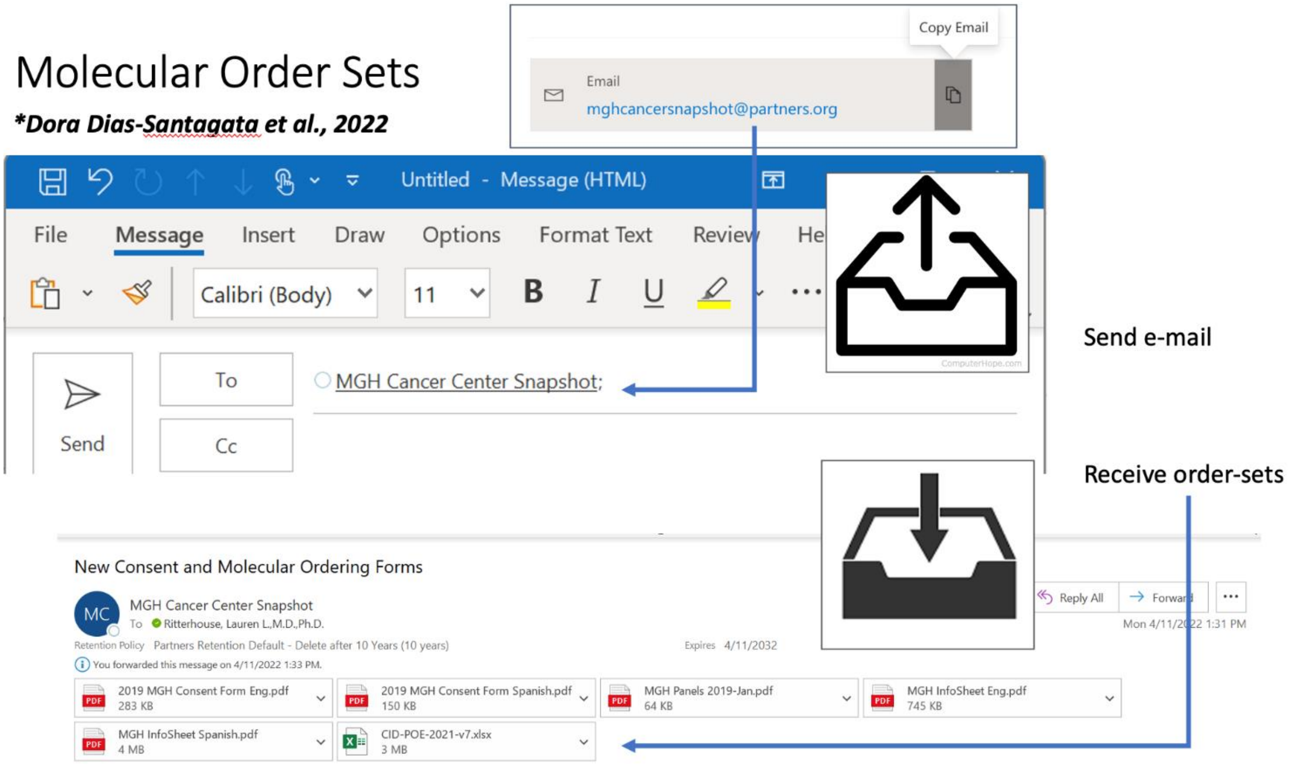


**Supplemental Figure 3. Molecular order sets retrieval.** Submit a blank email to: mghcancersnapshot@partners.org to obtain the most updated version of the order sets.

**Supplemental Table 4.** Non-recommended tests with potentially actionable findings.

| Test | Relevant Result | Variant Classification[1] | Therapy Given | Tumor Type | Sex | Period[2] | Added Value | Explanation |
|---|---|---|---|---|---|---|---|---|
| *EGFR* FISH | *EGFR* amplification | II-C | No | Esophagogastric | M | pre | Y | *EGFR* gain not detected by concurrent NGS |
| | | | No | Esophagogastric | M | pre | N | concurrent NGS POSITIVE for *EGFR* gain |
| | | | No | Colorectal | F | pre | N | concurrent NGS POSITIVE for *EGFR* gain |
| | | | No | Colorectal | M | post | N | concurrent NGS POSITIVE for *EGFR* gain |
| *FGFR1* FISH | *FGFR1* amplification | II-C | No | Biliary | F | post | Y | *FGFR1* gain not detected by concurrent NGS[3] |
| | | | No | Biliary | M | post | Y | *FGFR1* gain not detected by concurrent NGS |
| *HER2* FISH | *HER2/ERBB2* amplification | II-C | No | Pancreas | F | pre | Y | *ERBB2* gain not detected by concurrent NGS[3] |
| | | | No | Pancreas | F | pre | Y | NGS testing not requested |
| | | | No | Pancreas | F | pre | Y | *ERBB2* gain not detected by concurrent NGS[3] |
| | | I-B | No | Colorectal | F | pre | Y | *ERBB2* gain not detected by concurrent NGS[3] |
| | | | No | Colorectal | M | pre | Y | *ERBB2* gain not detected by concurrent NGS[3] |
| | | | No | Colorectal | M | pre | Y | *ERBB2* gain not detected by concurrent NGS[3] |
| | | | Yes (off-label) | Colorectal | F | Post | N | concurrent NGS POSITIVE for *ERBB2* gain |
| | | | No | Colorectal | M | Post | Y | *ERBB2* gain not detected by concurrent NGS[3] |
| *MET* FISH | *MET* amplification | II-C | No | Pancreas | M | pre | Y | *MET* gain not detected by concurrent NGS[3] |
| | | | No | Pancreas | M | pre | Y | *MET* gain not detected by concurrent NGS[3] |
| NGS: Snapshot | *CDKN2A* H83Y | II-D | No | Liver | M | pre | Y | NGS testing not in order set |
| | *IDH1* R132C | I-B | No | Liver (stage 4) | M | pre | n/a | upon closer evaluation these patients were found to have metastatic disease at the time of testing, therefore NGS-based analysis was indicated |
| | *CDK4* gain/amplification | II-C | No | Small intestine (stage 4) | M | pre | | |
| | *KRAS* G12C | II-C | No | Small intestine (stage 4) | M | pre | | |
| | *KRAS* G12V | II-C | No | Small intestine (stage 4) | M | pre | | |
| | *MAP2K1* K57E | II-C | Yes (clinical trial) | Small intestine (stage 4) | F | pre | | |
| | *PIK3CA* E545K | II-C | No | Small intestine (stage 4) | F | pre | | |
| | *CDK4* gain/amplification | II-C | No | Small intestine (stage 4) | M | post | | |
| | *ERBB2* gain/amplification | II-C | No | Small intestine (stage 4) | M | post | | |
| | *KRAS* G12A | II-C | No | Small intestine (stage 4) | F | post | | |
| | *KRAS* G12D | II-C | No | Small intestine (stage 4) | F | post | | |
| | *KRAS* Q61H | II-C | No | Small intestine (stage 4) | M | post | | |
| | *BRAF* G469A | II-C | Yes (clinical trial) | Small intestine (stage 4) | F | post | | |
| | *PIK3CA* R88Q | II-C | No | | | | | |
| | *ERBB2* S310F | II-C | No | Small intestine (stage 4) | M | post | | |
| | *KRAS* G12A and A146T | II-C | No | | | | | |

[1] Variant Tiers based on previously published guidelines. (Reference: Li MM, Datto M, Duncavage EJ, et al. Standards and guidelines for the interpretation and reporting of sequence variants in cancer: A joint consensus recommendation of the association for molecular pathology, American Society of Clinical Oncology, and College of American Pathologists. J Mol Diagn 2017;19:4-23)
[2] Refers to the two-year time frame used for clinical impact analysis, consisting of 1 year before and 1 year after roll-out of the GI order set.
[3] Concurrent NGS-based testing (Snapshot) was negative for copy number gains; however, the FISH results were consistent with low-level gene amplification and/or positive results in a small fraction of tumor cells (focal amplification).

## Supplemental Appendix 3. Analysis of actionable findings

Further analysis of the 30 non-recommended GI tests with potentially actionable findings (Supplemental Table 4) showed that, out of 16 gene amplifications identified by FISH, 4 (25%) were redundant findings, as recommended NGS testing of the tumor detected the same gain. Moreover, in 9 (56%) cases, positive FISH results were consistent with focal and/or low-level gene amplification, which may partially explain why concurrent NGS testing was negative for copy number gains (Table S3). Surprisingly, we found that 13 (43%) patients with actionable results, including the three patients who were treated based on molecular findings, had metastatic disease at the time of testing, and therefore qualified for NGS-based tumor profiling. In the end, these 13 requests were compatible with order set recommendations because the initial stratification at the time of ordering did not accurately reflect the true clinical indication (e.g. molecular testing on a complete excision of a small intestinal tumor in a patient who was ultimately stage IV) (Table S3 and checkmarks in Figure S2B). In the end, non-recommended tests uncovered novel, actionable findings (i.e. not detected by concurrent recommended testing) in 13 cases (n=10, 0.8% before, n=3, 0.2% after) and none of those patients received treatment as a result.

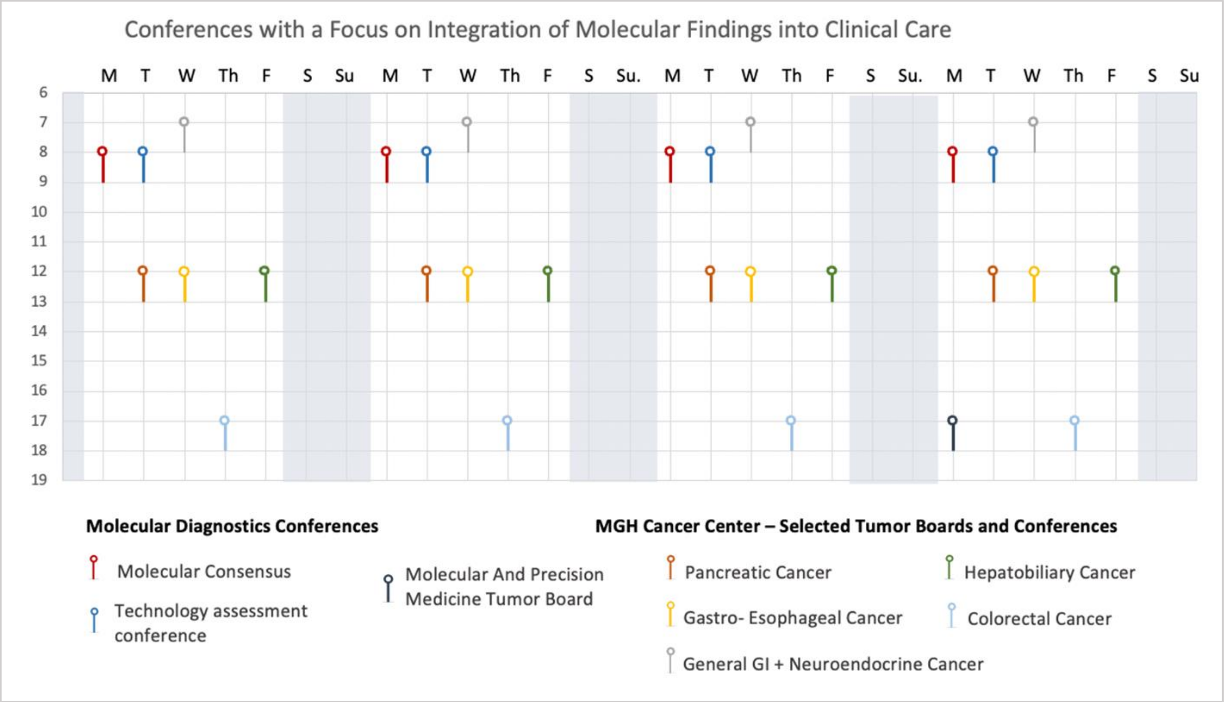


**Supplemental Figure 4. Representative monthly schedule for Molecular Diagnostics and GI oncology conferences.** These longstanding conferences include providers across the network and have remained essentially unchanged over the time frame covered by our study.
