# Supplementary material for: Implementation and Clinical Adoption of Precision Oncology Workflows Across a Healthcare Network
Source: Oncologist. 2022 Jul 19;27(11):930–9. doi: 10.1093/oncolo/oyac134 (PMC9632318; doi:10.1093/oncolo/oyac134)

Molecular Diagnostics POE Requisition (2021 version)

PLEASE SEND COMPLETED REQUISITION AND SIGNED CONSENT FORM TO MGHCANCERSNAPSHOT@PARTNERS.ORG

**Requesting Physician:**		**CC:**	
**Patient Name:**		**MRN:**	
**Diagnosis:**			
**Specimen Institution:**			
**Specimen #:**			
**Date of Surgery:**			
**Comments / Notes:**			

For all patients with a main provider in the Partners Healthcare Network, Informed written consent has to be obtained.
For all other requests please click/attest below

☐ "I attest that appropriate prior informed written consent has to be obtained"

https://malegislature.gov/Laws/GeneralLaws/PartI/TitleXVI/Chapter111/Section70G

FISH: Copy Number / Rearrangement

☐ ALK	☐ EWSR1	☐ cMYC
☐ ROS1	☐ DDIT3 (CHOP)	☐ BCL2
☐ RET	☐ SS18 (SYT)	☐ BCL6
☐ EGFR	☐ FOXO1 (FKHR)	
☐ MET	☐ FGFR1	
☐ HER2	☐ 1p19q	
☐ PDGFRA	☐ PIK3CA	
☐ CDKN2A		

NGS and PCR-Based Genotyping Assays

☐ NGS Solid Tumor Snapshot (SNVs/InDels/CNVs: incl. EGFR, BRAF, KRAS, NRAS, IDH1/2, MET, KIT, PDGFRA, TERT) & Solid Tumor Fusion Assay (Translocations: incl. ALK, ROS1, NTRK1/2/3, RET, MET, FGFR1/2/3, BRAF, NUTM1, MAML2, NRG1)

☐ NGS Sarcoma Fusion Assay (Translocations: incl. EWSR1, FUS, SS18, STAT6, CIC, NTRK3, PLAG1, USP6, JAZF1)

☐ MGMT	☐ Rapid EGFR
☐ MLH1	☐ Rapid BRAF
☐ MSI by IHC	☐ Rapid IDH1/IDH2
☐ MSI by PCR *(requires normal tissue or blood)*	

☐ **Panel Testing (content by site - see tabs)**
Please Review the Order Sets

Please provide the following pieces of information when requesting NGS-testing, otherwise the test cannot be performed

**Payor**	**Organ**	**Stage**

MGH CID – Lung Molecular Order Sets (2021.v4)

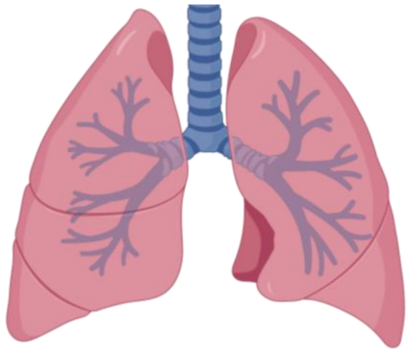

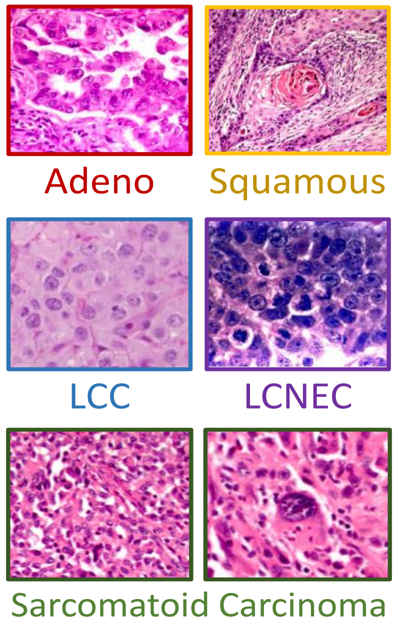

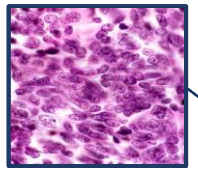

SCLC

**NSCLC**	Stage IA		N/A
	Stage IB-IIIA		NGS: Snapshot + Solid Fusion
			PD-L1/CD8 IHC (Stage II-III)
	Stage IIIB<br>Stage IIIC<br>Stage IV	never/minimal smoker	rapid *EGFR* + rapid Solid Fusion Assay
			ALK IHC => confirmatory Solid Fusion Assay or *ALK* FISH
			ROS1 IHC => confirmatory Solid Fusion Assay or *ROS1* FISH
			PD-L1/CD8 IHC
			NGS: Snapshot + Solid Fusion Assay (if not previously performed)
		smoker	PD-L1/CD8 IHC
			NGS: Snapshot + Solid Fusion Assay
		If no tissue available or FFPE exhausted	NGS: cf Lung
	Retesting after progression on	ALK-TKI	*MET* FISH
			NGS: Snapshot + Solid Fusion Assay
		EGFR-TKI	*MET* FISH
			EGFR FISH
			NGS: Snapshot + Solid Fusion Assay
		MET-TKI	*MET* FISH
			EGFR FISH
			NGS: Snapshot + Solid Fusion Assay
		ROS1-TKI	*MET* FISH
			NGS: Snapshot + Solid Fusion Assay
		other?	NGS: Snapshot + Solid Fusion Assay
		if no tissue available or FFPE exhausted	NGS: cfDNA Lung
**SCLC**	Stage (any)	never/minimal smoker	NGS: Snapshot + Fusion Assay

Tests in RED are <u>not</u> performed at the MGH CID and require a separate order (provided as best-practice recommendations)

MGH Cancer Center (Rebecca Heist, MD, Ibiayi Dagogo-Jack, MD); Pathology (Mari Mino-Kenudson, MD); Center for Integrated Diagnostics (Dora Dias-Santagata, PhD, FACMG, Jochen Lennerz, MD, PhD)

MGH CID – GI Molecular Order Sets (2021.v3)

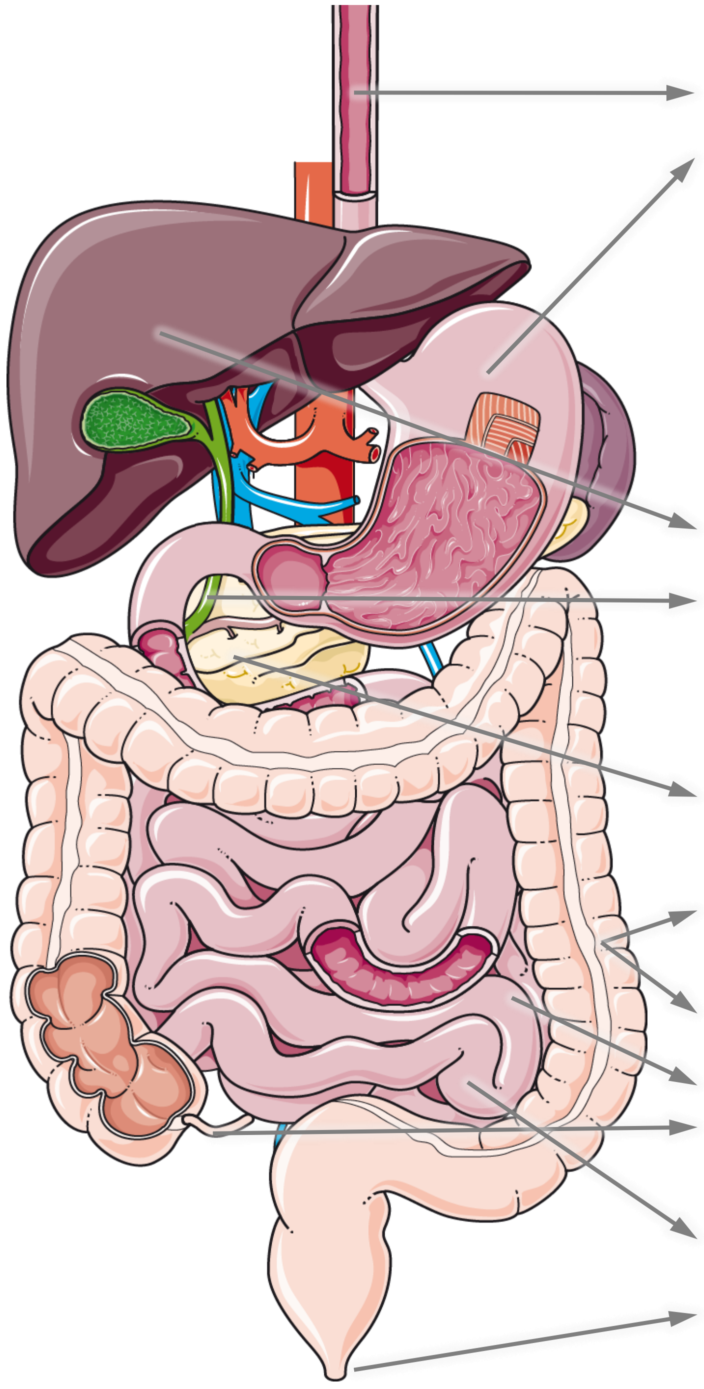

**Gastric or GEJ Adenoca:**	Stage I-III	HER2-IHC => if 2+ reflex *HER2* FISH<br>MMR IHC
	Stage IV	HER2-IHC => if 2+ reflex *HER2* FISH<br>*MET* FISH<br>NGS: Snapshot + Solid Fusion assay<br>PD-L1 IHC<br>MMR IHC<br>EBV (EBER-ISH) => order in appropriate setting
**Eso Squamous:**	Stage IV	HER2 IHC<br>PD-L1 IHC
**Liver:**	Stage IV	no routine testing recommended; (rarely Snapshot)
**Biliary:**	Stage IV	MMR IHC<br>PD-L1 IHC<br>NGS: Snapshot + Solid Fusion Assay<br>(*HER2*, *MET* FISH in small samples or low tumor purity)
**Pancreas:**	Stage IV	NGS: Snapshot + Solid Fusion Assay<br>MMR IHC
**Colorectal**	Stage I	MMR IHC => if abnormal refer to Lynch syndrome screening worksheet
	Stage II/III	CDX-2 IHC<br>MMR IHC => if abnormal refer to Lynch syndrome screening worksheet
**Colorectal<br>Small bowel<br>Appendix**	Stage IV	MMR IHC => if abnormal refer to Lynch syndrome screening worksheet<br>NGS: Snapshot + Solid Fusion Assay<br>*HER2* amp by NGS => confirmatory *HER2* FISH
**Carcinoid:**	Stage IV	NGS: Snapshot
**Anal Ca:**	Stage IV	PD-L1 IHC<br>HPV: either p16(IHC) or HPV(ISH)

Tests in RED are not performed at the MGH CID and require a separate order (provided as best-practice recommendations)

MGH Cancer Center (Jill Allen, MD; Jeffrey Clark, MD; Lipika Goyal, MD; Aparna Parikh, MD); Pathology (Joseph Misdraji, MD); Center for Integrated Diagnostics (Jochen Lennerz, MD, PhD)

MGH CID – Integrated Neuropathological Workup (2021.v1)

These workflows are NOT generating test orders.
Workflow and tests are provided as best-practice recommendations.

Diagnostic workup (Neuropathology) and patient management (Neuro-Oncology)

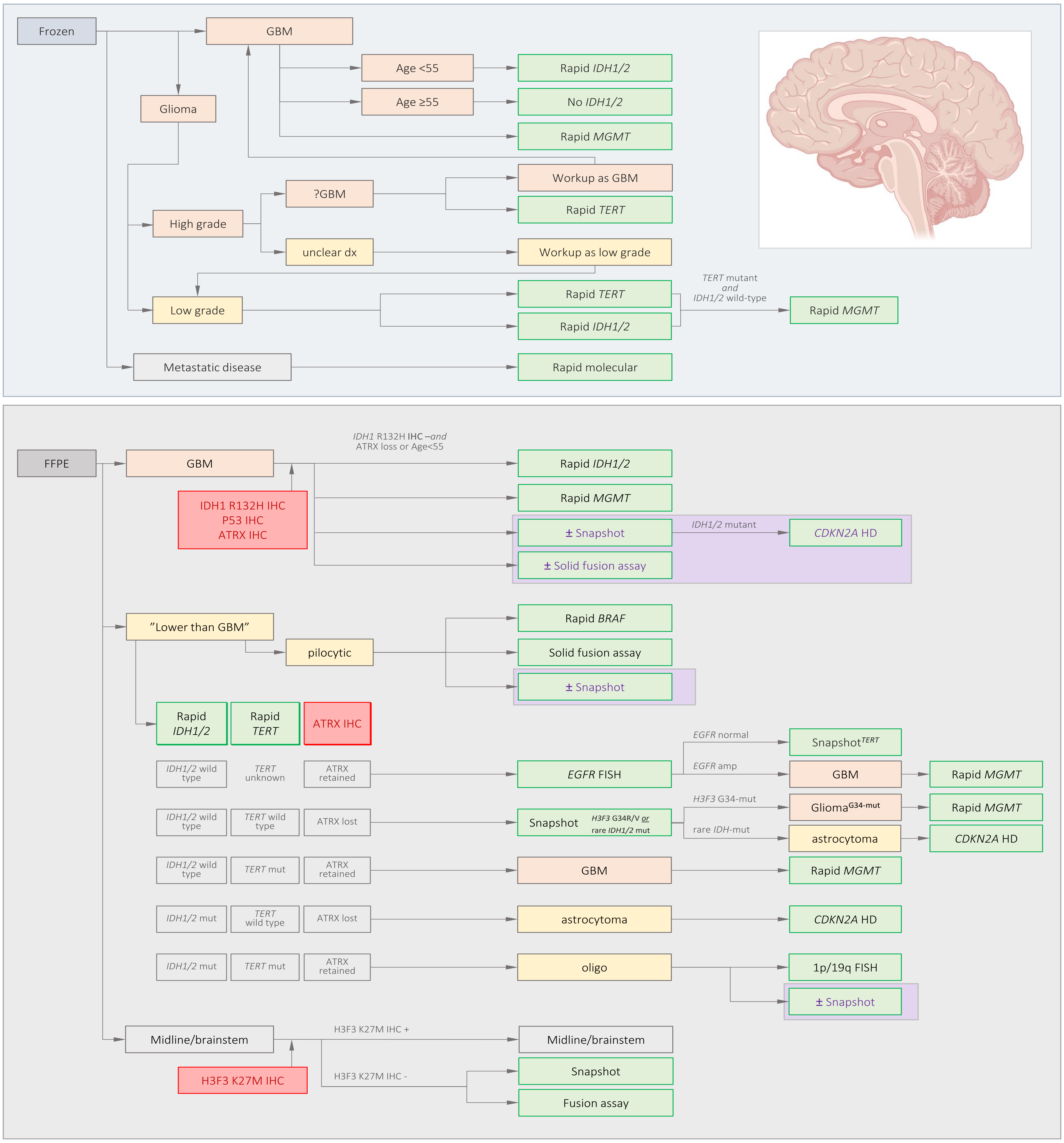

- diagnosed or suspected low-grade glioma
- diagnosed or suspected of high-grade glioma
- test routinely performed at the MGH Center for Integrated Diagnostics (CID)
- test typically requested by oncology, for patient management purposes
- tests in RED are *not* performed at the MGH CID and require a separate order (provided as best-practice recommendations)

MGH Cancer Center (Ganesh Shankar, MD, PhD; Justin Jordan, MD, MPH) ; Pathology (Maria Martinez-Lage Alvarez, MD; Matthew Frosch, MD, PhD)
Center for Integrated Diagnostics (Dora Dias-Santagata, PhD, FACMG; Jochen Lennerz, MD, PhD)

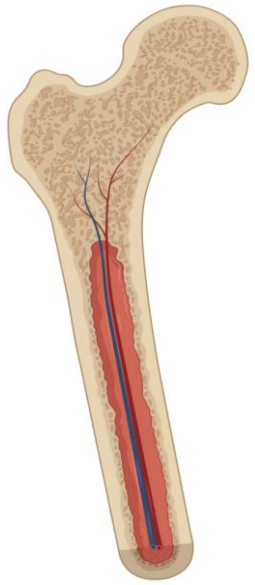

MGH CID – Myeloid Malignancies Molecular Order Sets (2021.v1)

at diagnosis

**AML**	Rapid testing: *NPM1/FLT3* ITD + *IDH1/2*
	Rapid Karyotype
	NGS: Heme Snapshot + Heme Fusion
**ALL**	Rapid *BCR-ABL1* (quantitiative RT-PCR)
	Karyotype
	ALL FISH panel (only for Pediatric ALL)(1)
	NGS: Heme Snapshot + Heme Fusion
	clonoSEQ assay of baseline specimen(2)
**Myeloproliferative neoplasm**	*JAK2/CALR* single gene assay (for blood)
	NGS: Heme Snapshot (for bone marrow)
	NGS: Heme Fusion (if snapshot is negative)
	BCR-ABL1 (quantitiative RT-PCR)
	Karyotype
**Myelodysplastic syndrome**	Karyotype
	NGS: Heme Snapshot
**Hyperosinophilia**	Karyotype
	NGS: Heme Snapshot + Heme Fusion

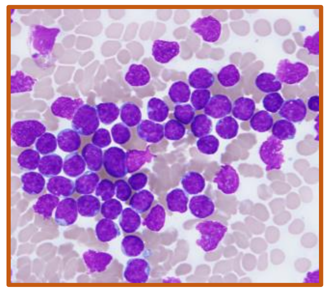

ALL

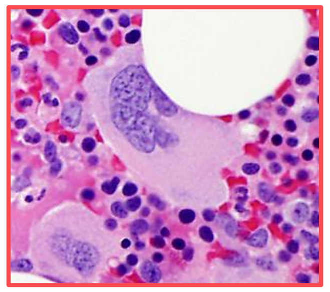

MPN

Tests in RED are not performed at the MGH CID and require a separate order (provided as best-practice recommendations)

(1) The FISH panel for pediatric ALL is available at the BWH CAMD Cytogenetics lab (https://www.brighamandwomens.org/assets/BWH/pathology/pdfs/bone-marrow-leukemia-req.pdf)

(2) The clonoSEQ assay is available at Adaptive Biotechnologies (additional information: clonoSEQ Clonality (ID) Test, https://www.clonoseq.com/for-clinicians/ordering/)

MGH Cancer Center (Timothy Graubert, MD; Amir Fathi, MD, Gabriella Hobbs, MD); Pathology (Robert Hasserjian, MD); Center for Integrated Diagnostics (Dora Dias-Santagata, PhD; Valentina Nardi, MD)

MGH CID – Lymphoid Malignancies Molecular Order Sets (2021.v1)

at diagnosis

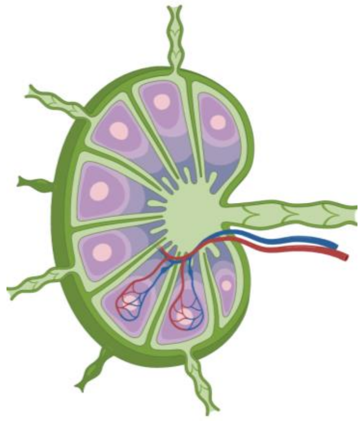

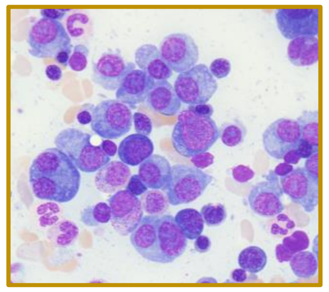

PCN (myeloma)

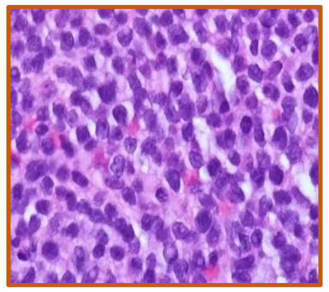

Lymphoma

**Plasma cell neoplasm**	NGS: Heme Snapshot => if negative reflex Solid Fusion Assay
	Diagnostic FISH panel for plasma cell proliferative disorders (1)
	Karyotype (only if patient also presents with unexplained cytopenia)
**Small B cell lymphoma**	NGS: Heme Snapshot
	CLL FISH panel (2)
	Karyotype, B-cell mitogen
**DLBCL**	*BCL2/BCL6/MYC* FISH (for gene rearrangements)
	Karyotype, B-cell mitogen
**T cell lymphoma**	Karyotype, T-cell mitogen
	NGS: Heme Snapshot + Heme Fusion

Tests in RED are not performed at the MGH CID and require a separate order (provided as best-practice recommendations)

(1) The FISH panel for plasma cell proliferative disorders is available at the Mayo Clinic (https://www.mayocliniclabs.com/test-catalog/Overview/606079)

(2) The CLL FISH panel is available at the BWH CAMD Cytogenetics laboratory (https://www.brighamandwomens.org/assets/BWH/pathology/pdfs/bone-marrow-leukemia-req.pdf)

MGH Cancer Center (Timothy Graubert, MD; Noopur Raje, MD; Jeremy Abramson, MD); Pathology (Robert Hasserjian, MD); Center for Integrated Diagnostics (Dora Dias-Santagata, PhD; Valentina Nardi, MD)

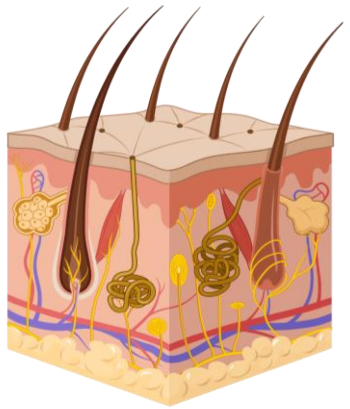

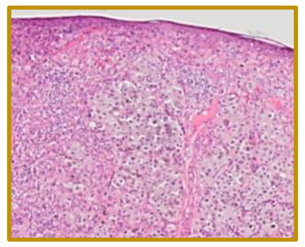

Melanoma

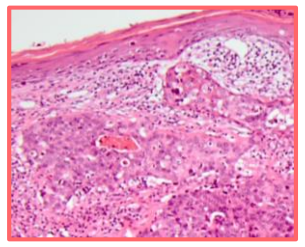

Sebaceous ca.

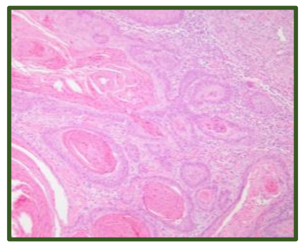

SCC

MGH CID – Skin Cancer Molecular Order Sets (2021.v2)

**Melanoma**	Stage I-II	N/A
	Stage III	BRAF V600E IHC (VE1)
		NGS: Snapshot + Solid Fusion Assay
	Stage IV	BRAF V600E IHC (VE1)
		rapid BRAF (PCR-based BRAF V600 mutation assay)
		NGS: Snapshot + Solid Fusion Assay
**Merkel Cell Carcinoma**	Stage (any)	Merkel Cell Polyoma Virus IHC + Serum Antibody Test (1)
	Stage II-IV	NGS: Snapshot + Solid Fusion Assay
**Sebaceous Carcinoma**	Stage I-II	N/A
	Stage III-IV *metastatic or unresectable/morbidly resectable disease*	MMR IHC
		NGS: Snapshot + Solid Fusion Assay
**Others (Adnexal tumors, SCC, BCC, etc.)**	Stage I-II	N/A
	Stage III-IV *metastatic or unresectable/morbidly resectable disease*	NGS: Snapshot + Solid Fusion Assay

Tests in RED are not performed at the MGH CID and require a separate order (provided as best-practice recommendations)

(1) Please refer to the Merkel Cell Antibody order in Epic (additional information: https://merkelcell.org/testing-and-diagnosis/sero/)

MGH Cancer Center (Ryan Sullivan, MD; David Miller, MD, PhD); Pathology (Mai Hoang, MD); Center for Integrated Diagnostics (Dora Dias-Santagata, PhD, FACMG)

MGH CID – Breast Molecular Order Sets (2021.v2)

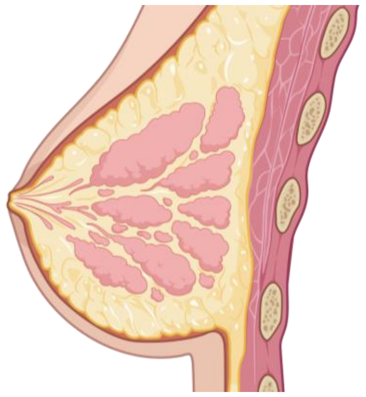

**Breast Cancer (all types)**	Stage (any)	Genetic counseling if patient is at risk for hereditary breast cancer [1]
**Invasive Breast Cancer**	Stage I-III	N/A
**Recurrent or Metastatic Breast Cancer**	Stage IV	*BRCA1 /BRCA2* germline sequencing
		MMR IHC
		NGS: Snapshot + Solid Fusion Assay
		FGFR1 FISH *(ad hoc)*
		PD-L1 IHC (for triple-negative breast cancer, reported w/ breast-specific scoring)
	If no tissue available or FFPE exhausted	NGS: cfDNA Breast
**Re-testing**	disease progression on therapy (>=6 months)	NGS: cfDNA Breast
**DCIS**		N/A

Tests in RED are not performed at the MGH CID and require a separate order (provided as best-practice recommendations)

(1) Genetic counseling requires a separate referral

MGH Cancer Center (Aditya Bardia, MD; Steven Isakoff, MD, PhD); Pathology (Amy Ly, MD); Center for Integrated Diagnostics (Dora Dias-Santagata, PhD, FACMG)

MGH CID – GYN Molecular Order Sets (2021.v3)

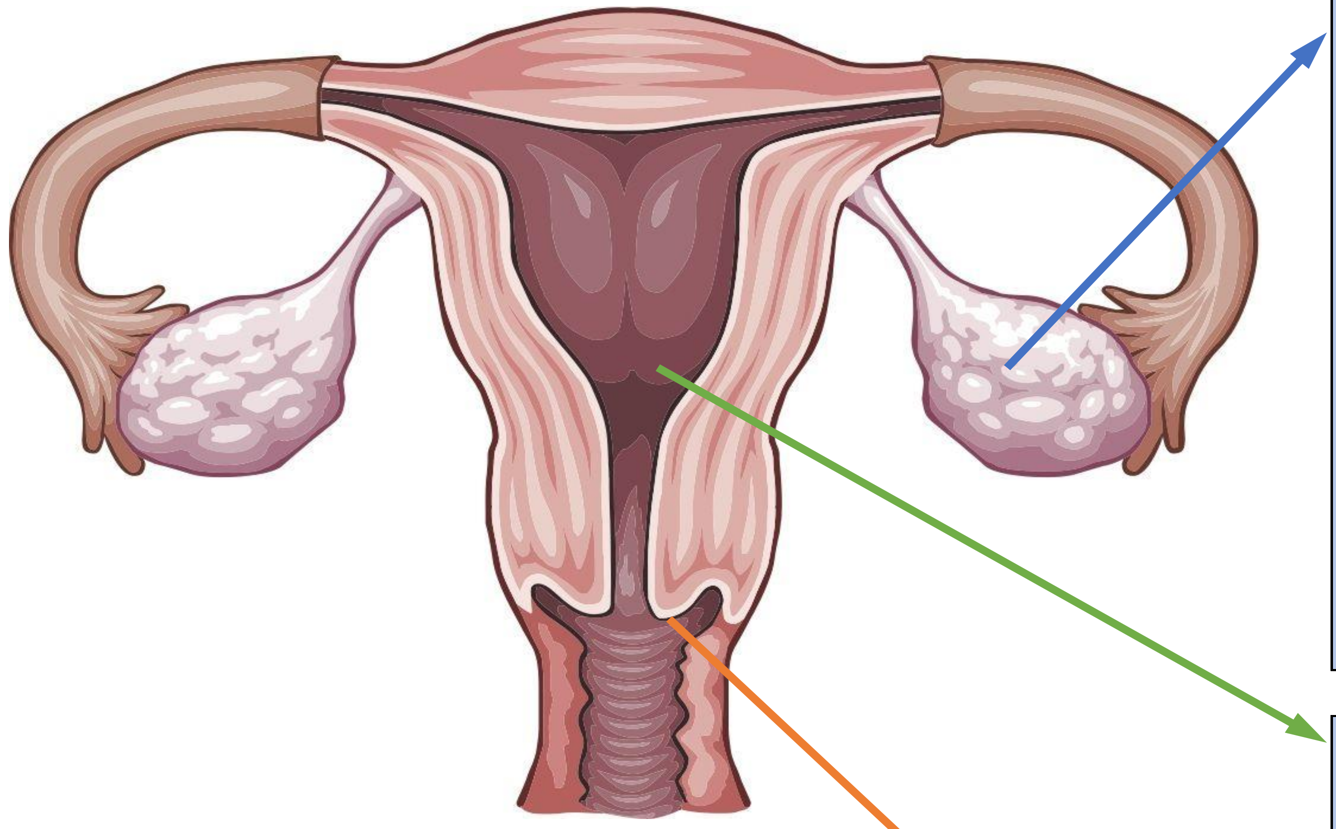

Organ	Subtype or Setting		Test
Ovary/Fallopian tube	serous	Stage I-II	Genetic counseling + Germline testing
	serous	Stage III-IV	Genetic counseling + Germline testing<br>HRD score
	endometrioid		Genetic counseling + Germline testing<br>MMR-IHC
	clear cell		Genetic counseling + Germline testing<br>MMR-IHC
	undifferentiated/dedifferentiated		Genetic counseling + Germline testing<br>MMR-IHC
	other, non-serous		Genetic counseling + Germline testing
	any carcinoma	recurrent	Genetic counseling + Germline testing; if not previously performed<br>MMR-IHC; if not previously performed<br>HRD score; if not previously performed<br>NGS: Snapshot + Solid Fusion Assay
Uterine corpus	carcinoma	all	TP53/MMR IHC
		carcinosarcoma	TP53/MMR IHC + HER2 IHC (reflex to *HER2* FISH for 2+ HER2 IHC)
		serous	TP53/MMR IHC + HER2 IHC (reflex to *HER2* FISH for 2+ HER2 IHC)
		high-grade endometrioid	TP53/MMR IHC + HER2 IHC (reflex to *HER2* FISH for 2+ HER2 IHC)
	Uterine tumor resembling ovarian sex cord tumor		Solid Fusion Assay (diagnostic)
	Inflammatory myofibroblastic tumor		Solid Fusion Assay (diagnostic)
	Leiomyosarcoma		Sarcoma Fusion (diagnostic)
	Rhabdomyosarcoma		Sarcoma Fusion (diagnostic)
	Endometrial stromal sarcoma		Solid Fusion Assay (diagnostic); if negative, reflex to Sarcoma Fusion
Cervix	squamous carcinoma	stage IV or recurrent	PD-L1/p16 IHC<br>NGS: Snapshot + Solid Fusion Assay
	adenocarcinoma	stage IV or recurrent	PD-L1/p16 IHC
Vagina/Vulva	squamous carcinoma	primary	p16/TP53 IHC
	squamous carcinoma	recurrent	PD-L1/p16/TP53 IHC<br>NGS: Snapshot + Solid Fusion Assay

Tests in RED are not performed at the MGH CID and require a separate order (provided as best-practice recommendations)
Of note, genetic counseling requires a separate referral, and HRD score + germline testing are available at Myriad Genetics (additional information: myChoiceCDx test, https://myriad-oncology.com/mychoice-cdx/, https://myriad-oncology.com/mychoice-cdx/providers/)

MGH Cancer Center (Sara Bouberhan, MD); Pathology (Jaclyn Watkins, MD, MS; Esther Oliva, MD); Center for Integrated Diagnostics (Jochen Lennerz, MD, PhD; Dora Dias-Santagata, PhD, FACMG; Lauren Ritterhouse, MD, PhD)

MGH CID – Endocrine Molecular Order Sets (2021.v1)

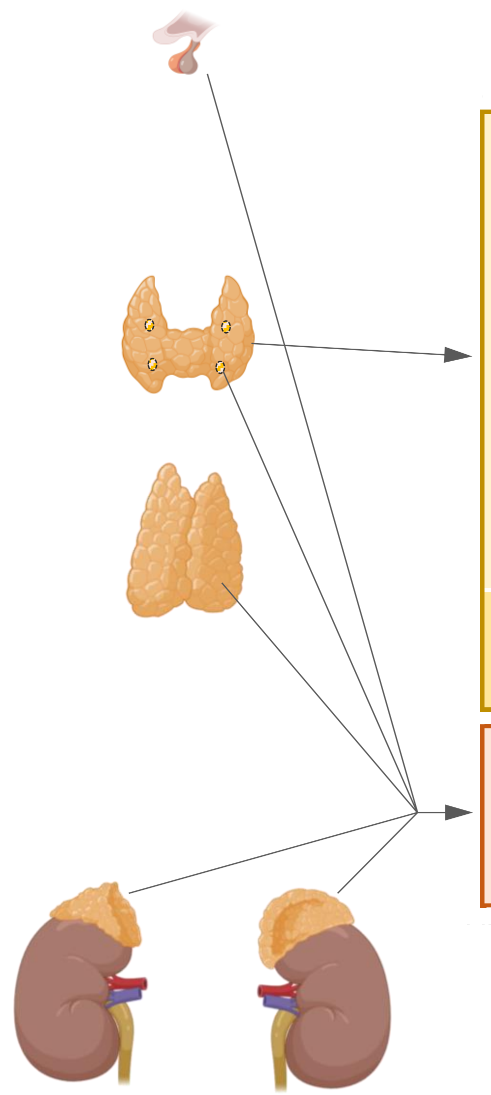

**Thyroid**	Medullary Thyroid Cancer	Stage (any)	Genetic counseling if germline testing has not been done (for patient or family) [1]
		Stage III-IV *distant mets or locally advanced/bulky disease*	NGS: Snapshot => if negative reflex Solid Fusion Assay
	Differentiated Thyroid Cancer	DTC with papillary features (any stage)	BRAF V600E IHC
		Radioiodine-refractory DTC	NGS: Snapshot + Solid Fusion Assay
	Anaplastic Thyroid Cancer	Stage (any)	BRAF V600E IHC
			NGS: Snapshot + Solid Fusion Assay
**Re-testing**	disease progression on therapy		NGS: Snapshot + Solid Fusion Assay (preferred)
			NGS: cfDNA NGS (if no tissue available or FFPE exhausted)
**Others (Parathyroid, Adrenal, Pituitary, etc)**		Stage I-III	N/A
		Stage IV	MMR IHC (Adrenal)
			NGS: Snapshot + Solid Fusion Assay

Tests in RED are not performed at the MGH CID and require a separate order (provided as best-practice recommendations)

(1) Genetic counseling requires a separate referral

MGH Cancer Center (Lori Wirth, MD); Pathology (Peter Sadow, MD, PhD); Center for Integrated Diagnostics (Dora Dias-Santagata, PhD, FACMG)

MGH CID – Head and Neck Molecular Order Sets (2021.v1)

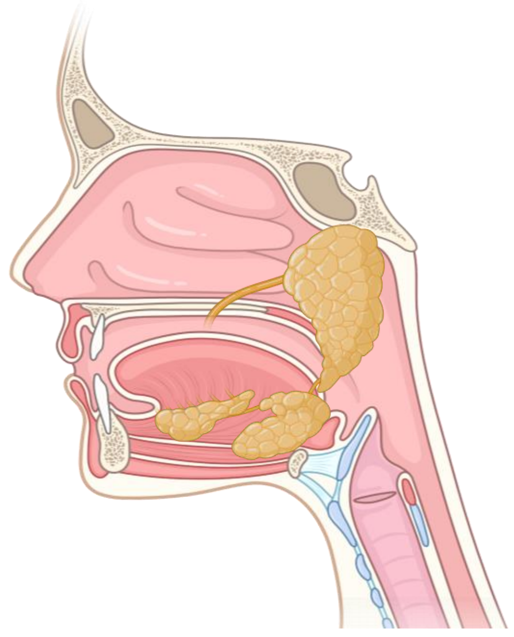

Patient management (Oncology)

**Salivary Glands**	Stage I-III	N/A
	Stage IV	PD-L1 IHC
		HER2 IHC => if 2+ reflex *HER2* FISH
		NGS: Snapshot + Solid Fusion Assay
**Nasopharynx**	Stage (any)	EBV-encoded RNA (EBER) *in-situ* hybridization (ISH) => if negative reflex HPV testing: p16 IHC and specific testing for high-risk (hr) HPV strains (ISH or PCR)
	Stage IV	PD-L1 IHC
		NGS: Snapshot + Solid Fusion Assay
**Oropharynx or Sinonasal SCC**	Stage (any)	HPV testing: p16 IHC and hrHPV-specific testing (ISH or PCR)
	Stage IV	PD-L1 IHC
		NGS: Snapshot + Solid Fusion Assay
**HNSCC/not Oropharynx or Sinonasal**	Stage I-III	N/A
	Stage IV	PD-L1 IHC
		NGS: Snapshot + Solid Fusion Assay
**Unknown Primary HNSCC**	Stage (any)	HPV testing: p16 IHC and hrHPV-specific testing (ISH or PCR)
	Stage IV	PD-L1 IHC
		NGS: Snapshot + Solid Fusion Assay

Diagnostically challenging cases (Pathology)

Salivary gland tumors

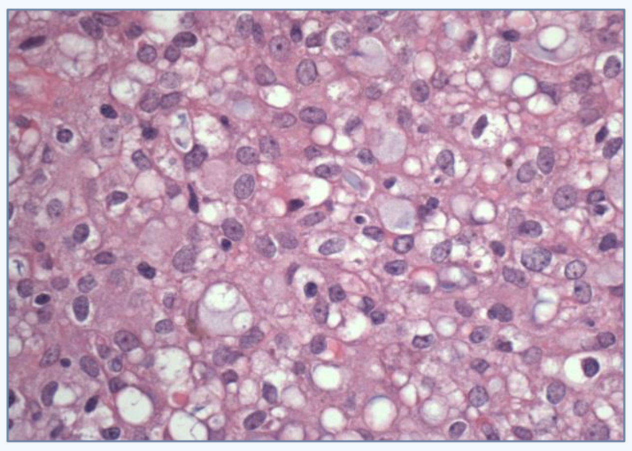

make dx/refine dx — Solid Fusion Assay

refine dx — Snapshot

Sinonasal salivary-type carcinoma

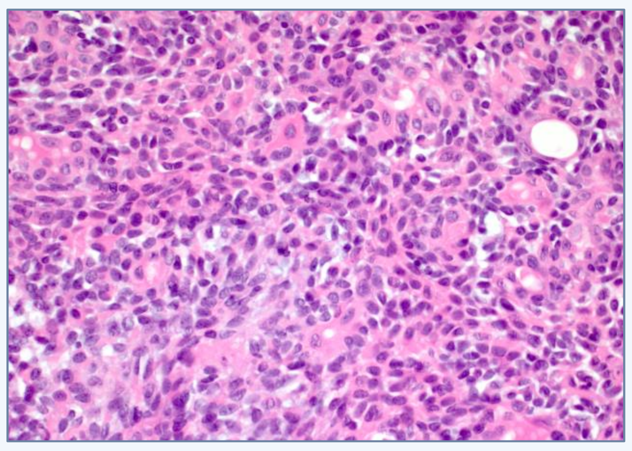

make dx — Solid Fusion Assay

make dx/refine dx — Snapshot

Sinonasal SCC

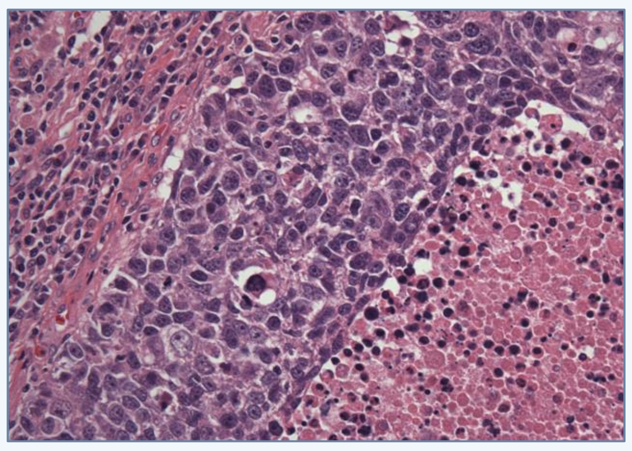

make dx/refine dx — Snapshot

Tests in RED are not performed at the MGH CID and require a separate order (provided as best-practice recommendations)

MGH Cancer Center (Lori Wirth, MD); Pathology (William Faquin, MD, PhD; Peter Sadow, MD, PhD); Center for Integrated Diagnostics (Dora Dias-Santagata, PhD, FACMG)

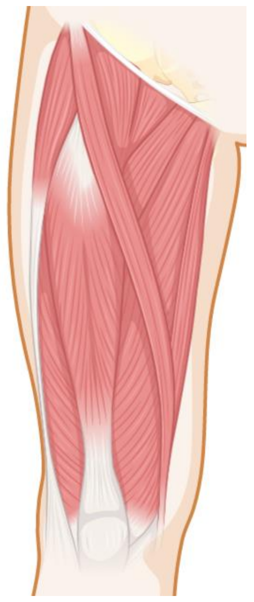

MGH CID – Bone and Soft Tissue Molecular Order Sets (2021.v2)

Patient management (Oncology)

**GIST**	Stage (any)	SDHB IHC
		PD-L1 IHC
		NGS: Snapshot + Solid Fusion Assay
**Sarcoma**	Stage I-II	N/A
	Stage III-IV *metastatic or unresectable/morbidly resectable disease*	PD-L1 IHC
		NGS: Snapshot + Solid Fusion Assay

Diagnostically challenging cases (Pathology)

Ewing / DSRCT	Synovial sarcoma	SFT	Non-descript *round, spindle, pleomorphic*	Liposarcoma *atypical lipomatous tumors*	Cartilaginous ?	Desmoid ?
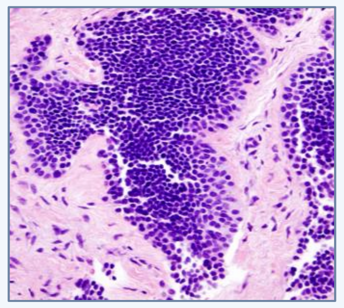	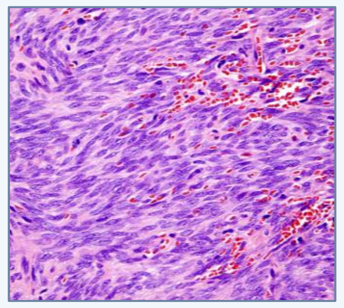	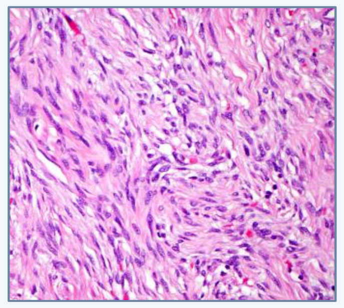	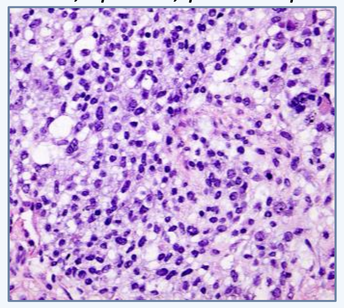	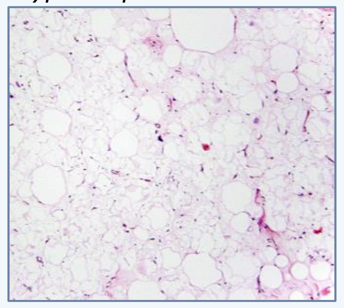	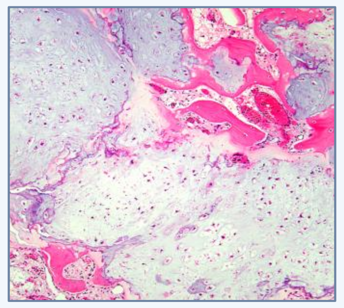	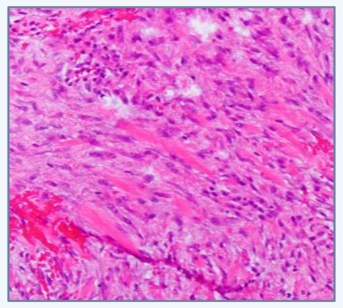
make dx: *EWSR1* FISH → if ⊝ → *FUS* FISH; refine dx: Sarcoma Fusion	make dx: *SS18* FISH; refine dx: Sarcoma Fusion	make dx: Sarcoma Fusion	make dx: Sarcoma Fusion → if ⊝ → Solid Fusion or Heme Fusion	make dx: *MDM2* FISH [(1)] → if ⊝ → Sarcoma Fusion	make dx: *IDH1/IDH2* mutation test	make dx: Snapshot [(2)] (*CTNNB1* or *APC* mut) → if ⊝ → Sarcoma Fusion

Tests in RED are not performed at the MGH CID and require a separate order (provided as best-practice recommendations)

(1) *MDM2* FISH is available at the BWH CAMD Cytogenetics laboratory (https://www.brighamandwomens.org/assets/BWH/pathology/pdfs/solid-tumor-lymph-node-req.pdf)

(2) Snapshot NGS testing requires patient consent

MGH Cancer Center (Gregory Cote, MD, PhD); Pathology (Yin Hung, MD, PhD); Center for Integrated Diagnostics (Dora Dias-Santagata, PhD, FACMG)

Molecular Pathology testing for the diagnosis of challenging bone and soft tissue tumors

Ewing sarcoma/DSRCT/other small round cell tumors	*EWSR1* FISH => if negative reflex to *FUS* FISH	to confirm morphological impression (only STAT cases, or if tissue is limiting)
	Sarcoma Fusion	to refine diagnosis/improve subclassification
Synovial sarcoma	*SS18* FISH	to confirm morphological impression (only STAT cases, or if tissue is limiting)
	Sarcoma Fusion	to refine diagnosis/improve subclassification
Solitary fibrous tumor (especially high-grade variants)	Sarcoma Fusion	for challenging cases: *STAT6* fusions => SFT vs. other rearrangements => help inform difficult diagnoses
Other non-descript round/spindle/pleomorphic sarcomas	Sarcoma Fusion => if negative consider Solid Fusion Assay or Heme Fusion	for challenging cases: choice of fusion assay depends on histomorphology, or suspicion/prior knowledge of a specific rearrangement
Well-differentiated/dedifferentiated liposarcoma and atypical lipomatous tumor	*MDM2* FISH[(1)] => if negative consider Sarcoma Fusion	assessment of *MDM2* gene amplification to confirm morphological impression/diagnose challenging cases
Chondrosarcoma and other cartilaginous tumors	*IDH* mutation testing	to assess if a tumor is of cartilaginous derivation: *IDH*^{mut} => chondrosarcoma/other cartilaginous tumors; *IDH*^{WT} => chondroblastic osteosarcoma/other sarcomas
Desmoid fibromatosis	Snaphot NGS[(2)] (*CTNNB1* or *APC* mutations) => if negative consider Sarcoma Fusion	to confirm morphological impression/diagnose challenging cases: *CTNNB1*^{mut} or *APC*^{mut} => desmoid fibromatosis; *CTNNB1*^{WT} or *APC*^{WT} => benign vs. another low grade sarcoma

MGH CID – GU Molecular Order Sets (2021.v2)

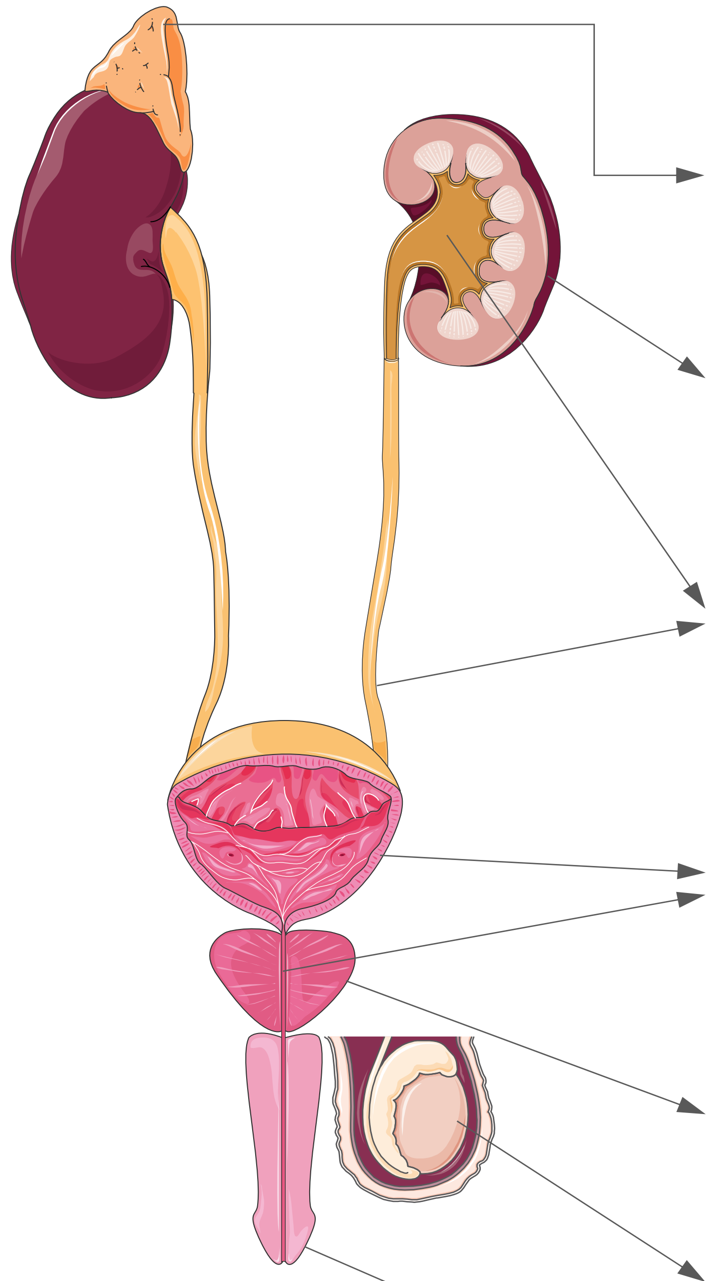

Others (Adrenal, Pheo, etc.)	Stage I-III	N/A
	Stage IV	MMR IHC (Adrenal)
		NGS: Snapshot + Solid Fusion Assay
Kidney	Stage I-III	N/A
	Stage IV	MMR IHC
		NGS: Snapshot + Solid Fusion Assay
Renal Pelvis/Ureter	Stage I-III	MMR IHC
	Stage IV	MMR IHC
		PD-L1 IHC
		NGS: Snapshot + Solid Fusion Assay
Bladder/Urethra	Stage I-III	N/A
	Stage IV	MMR IHC
		PD-L1 IHC
		NGS: Snapshot + Solid Fusion Assay
Prostate	Stage I-III	N/A
	Stage IV	MMR IHC
		NGS: Snapshot + Solid Fusion Assay
Testicular	Stage I-III	N/A
	Stage IV	NGS: Snapshot + Solid Fusion Assay
Penile squamous cell carcinoma	Stage (any)	p16 IHC
		HPV *in-situ* hybridization (ISH) or RT-PCR

Tests in RED are not performed at the MGH CID and require a separate order (provided as best-practice recommendations)

MGH Cancer Center (Xin Gao, MD); Pathology (Chin-Lee Wu, MD, PhD); Center for Integrated Diagnostics (Dora Dias-Santagata, PhD, FACMG)

Lynch syndrome screening worksheet for Colorectal Cancer

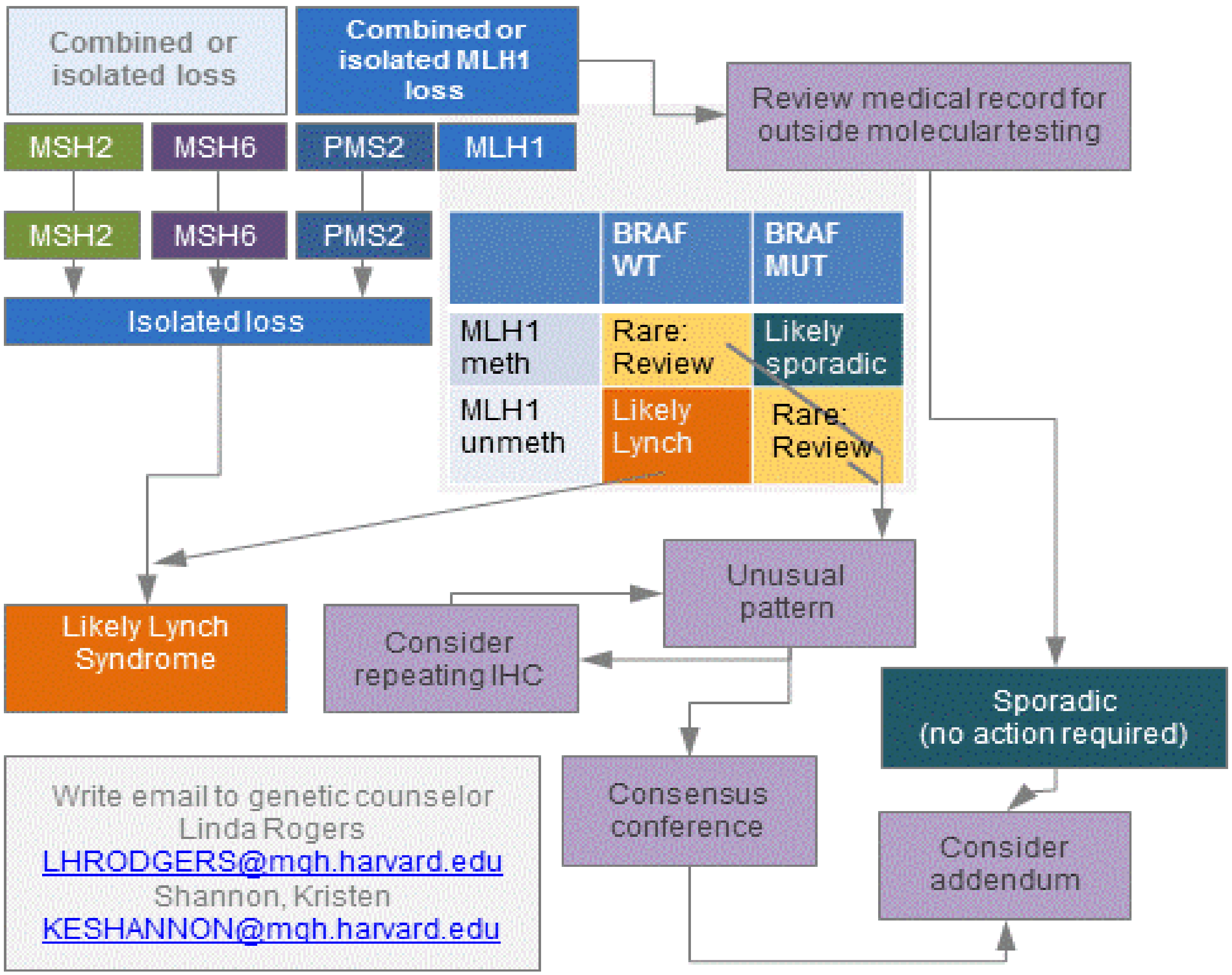

Lynch syndrome screening worksheet for Uterine Cancer

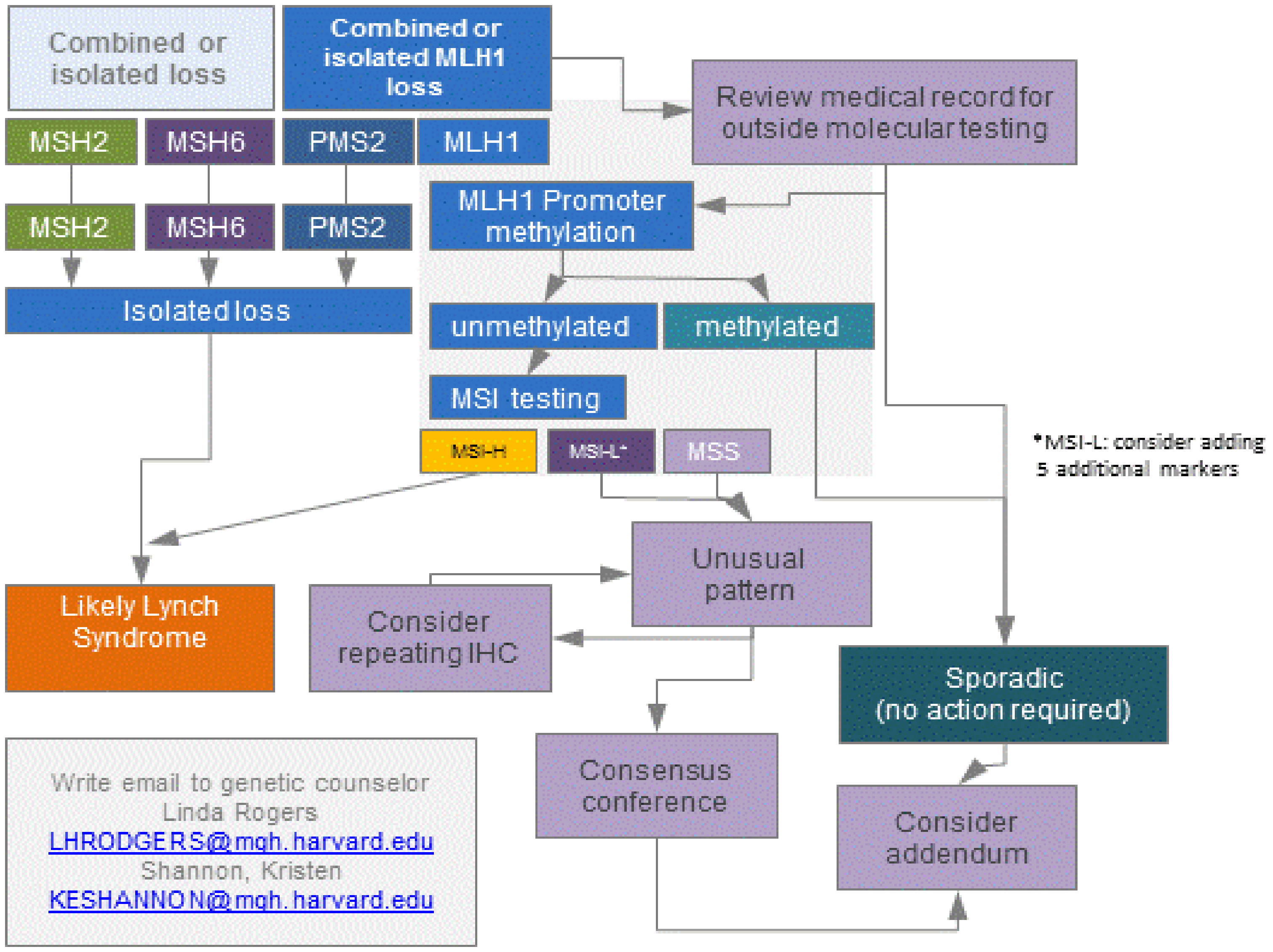

Supplement: oyac134_suppl_Supplementary_Information_2 [file oyac134_suppl_supplementary_information_2.pdf]
